# Expression of γ-globin genes in β-thalassemia patients treated with sirolimus: results from a pilot clinical trial (Sirthalaclin)

**DOI:** 10.1177/20406207221100648

**Published:** 2022-06-21

**Authors:** Cristina Zuccato, Lucia Carmela Cosenza, Matteo Zurlo, Jessica Gasparello, Chiara Papi, Elisabetta D’Aversa, Giulia Breveglieri, Ilaria Lampronti, Alessia Finotti, Monica Borgatti, Chiara Scapoli, Alice Stievano, Monica Fortini, Eric Ramazzotti, Nicola Marchetti, Marco Prosdocimi, Maria Rita Gamberini, Roberto Gambari

**Affiliations:** Dipartimento di Scienze della Vita e Biotecnologie, Sezione di Biochimica e Biologia Molecolare, Università degli Studi di Ferrara, Ferrara, Italy; Dipartimento di Scienze della Vita e Biotecnologie, Sezione di Biochimica e Biologia Molecolare, Università degli Studi di Ferrara, Ferrara, Italy; Dipartimento di Scienze della Vita e Biotecnologie, Sezione di Biochimica e Biologia Molecolare, Università degli Studi di Ferrara, Ferrara, Italy; Dipartimento di Scienze della Vita e Biotecnologie, Sezione di Biochimica e Biologia Molecolare, Università degli Studi di Ferrara, Ferrara, Italy; Dipartimento di Scienze della Vita e Biotecnologie, Sezione di Biochimica e Biologia Molecolare, Università degli Studi di Ferrara, Ferrara, Italy; Dipartimento di Scienze della Vita e Biotecnologie, Sezione di Biochimica e Biologia Molecolare, Università degli Studi di Ferrara, Ferrara, Italy; Dipartimento di Scienze della Vita e Biotecnologie, Sezione di Biochimica e Biologia Molecolare, Università degli Studi di Ferrara, Ferrara, Italy; Dipartimento di Scienze della Vita e Biotecnologie, Sezione di Biochimica e Biologia Molecolare, Università degli Studi di Ferrara, Ferrara, Italy; Thal-LAB, Laboratorio di Ricerca Elio Zago sulla Terapia Farmacologica e Farmacogenomica della Talassemia, Università degli Studi di Ferrara, Ferrara, Italy; Dipartimento di Scienze della Vita e Biotecnologie, Sezione di Biochimica e Biologia Molecolare, Università degli Studi di Ferrara, Ferrara, Italy; Thal-LAB, Laboratorio di Ricerca Elio Zago sulla Terapia Farmacologica e Farmacogenomica della Talassemia, Università degli Studi di Ferrara, Ferrara, Italy; Dipartimento di Scienze della Vita e Biotecnologie, Sezione di Biochimica e Biologia Molecolare, Università degli Studi di Ferrara, Ferrara, Italy; Thal-LAB, Laboratorio di Ricerca Elio Zago sulla Terapia Farmacologica e Farmacogenomica della Talassemia, Università degli Studi di Ferrara, Ferrara, Italy; Dipartimento di Scienze della Vita e Biotecnologie, Sezione di Biologia ed Evoluzione, Università degli Studi di Ferrara, Ferrara, Italy; Unità Operativa Interdipartimentale di Day Hospital della Talassemia e delle Emoglobinopatie, Arcispedale S. Anna di Ferrara, Ferrara, Italy; Unità Operativa Interdipartimentale di Day Hospital della Talassemia e delle Emoglobinopatie, Arcispedale S. Anna di Ferrara, Ferrara, Italy; Laboratorio Unico Metropolitano, Ospedale Maggiore, Azienda USL di Bologna, Bologna, Italy; Dipartimento di Scienze Chimiche, Farmaceutiche e Agrarie, Università degli Studi di Ferrara, Ferrara, Italy; Rare Partners S.r.L. Impresa Sociale, Milano, Italy; Unità Operativa Interdipartimentale di Day Hospital della Talassemia e delle Emoglobinopatie, Arcispedale S. Anna di Ferrara, via Aldo Moro, 8, Ferrara 44124, Italy; Dipartimento di Scienze della Vita e Biotecnologie, Sezione di Biochimica e Biologia Molecolare, Università degli Studi di Ferrara, via Fossato di Mortara, 74, Ferrara 44121, Italy; Thal-LAB, Laboratorio di Ricerca Elio Zago sulla Terapia Farmacologica e Farmacogenomica della Talassemia, Università degli Studi di Ferrara, Ferrara, Italy; Center ‘Chiara Gemmo and Elio Zago’ for the Research on Thalassemia, Università degli Studi di Ferrara, Ferrara, Italy

**Keywords:** β-thalassemia, fetal hemoglobin, γ-globin mRNA, ineffective erythropoiesis, sirolimus, transfusion requirement

## Abstract

**Introduction:**

β-thalassemia is caused by autosomal mutations in the β-globin gene, which induce the absence or low-level synthesis of β-globin in erythroid cells. It is widely accepted that a high production of fetal hemoglobin (HbF) is beneficial for patients with β-thalassemia. Sirolimus, also known as rapamycin, is a lipophilic macrolide isolated from a strain of *Streptomyces hygroscopicus* that serves as a strong HbF inducer *in vitro* and *in vivo*. In this study, we report biochemical, molecular, and clinical results of a sirolimus-based NCT03877809 clinical trial (a personalized medicine approach for β-thalassemia transfusion-dependent patients: testing sirolimus in a first pilot clinical trial, Sirthalaclin).

**Methods:**

Accumulation of γ-globin mRNA was analyzed using reverse-transcription quantitative polymerase chain reaction (PCR), while the hemoglobin pattern was analyzed using high-performance liquid chromatography (HPLC). The immunophenotype was analyzed using a fluorescence-activated cell sorter (FACS), with antibodies against CD3, CD4, CD8, CD14, CD19, CD25 (for analysis of peripheral blood mononuclear cells), or CD71 and CD235a (for analysis of *in vitro* cultured erythroid precursors).

**Results:**

The results were obtained in eight patients with the β^+^/β^+^ and β^+^/β^0^ genotypes, who were treated with a starting dosage of 1 mg/day sirolimus for 24–48 weeks. The first finding of this study was that the expression of γ-globin mRNA increased in the blood and erythroid precursor cells isolated from β-thalassemia patients treated with low-dose sirolimus. This trial also led to the important finding that sirolimus influences erythropoiesis and reduces biochemical markers associated with ineffective erythropoiesis (excess free α-globin chains, bilirubin, soluble transferrin receptor, and ferritin). A decrease in the transfusion demand index was observed in most (7/8) of the patients. The drug was well tolerated, with minor effects on the immunophenotype, and an only side effect of frequently occurring stomatitis.

**Conclusion:**

The data obtained indicate that low doses of sirolimus modify hematopoiesis and induce increased expression of γ-globin genes in a subset of patients with β-thalassemia. Further clinical trials are warranted, possibly including testing of the drug in patients with less severe forms of the disease and exploring combination therapies.

## Introduction

β-thalassemias are among the most common inherited hemoglobinopathies worldwide and are caused by autosomal mutations in the gene encoding β-globin, which cause an absence or low-level synthesis of this protein in erythropoietic cells.^1–3^ The phenotypes range from asymptomatic (β-thalassemia trait or carrier) to clinically relevant anemia, which is categorized as transfusion-dependent β-thalassemia (TDT, including thalassemia major) or non-transfusion-dependent β-thalassemia (NTDT, thalassemia intermedia).

Approximately, 80–90 million people (~1.5% of the global population) are carriers of β-thalassemia, with approximately 60,000 symptomatic individuals born annually.^4–7^ Worldwide, nearly 300 mutations in the β-globin gene have been reported to result in β-thalassemia.^8^ Most are point mutations, either in the gene or its immediate flanking regions, but some are caused by small deletions that remove all or part of the β-globin gene.

It is widely accepted that a high production of fetal hemoglobin (HbF) is beneficial for patients.^9–11^ The earliest clinical observations suggesting the advantageous role of HbF in thalassemia came from patients with rare forms of β-thalassemia, particularly those with large deletions responsible for δβ^0^-thalassemia or hereditary persistence of fetal hemoglobin (HPFH), which is characterized by the absence of β-globin production, but high levels of γ-globin chain production, resulting in high levels of HbF with a relatively benign clinical course.^12^ More recent clinical studies have shown that naturally higher production of HbF improves the clinical course of a variety of β-thalassemia patients.^13–17^ Accordingly, these observations have prompted research on inducers of HbF that can therapeutically reproduce what occurs in β-thalassemia patients, with natural persistence of higher levels of HbF.^18–21^ Furthermore, genome editing approaches are available for the induction of HbF following the elimination of genomic sequences encoding for transcriptional repressors or genomic sequences targeted by these regulatory factors.^22–25^ In this context, clinical trials are underway, such as NCT03655678 (A safety and efficacy study evaluating CTX001 in subjects with TDT), based on the use of autologous CRISPR-Cas9-modified CD34^+^ human hematopoietic stem and progenitor cells with CTX001.^26^

Sirolimus, also known as rapamycin,^27^ is a lipophilic macrolide isolated from a strain of *S. hygroscopicus* found in soil from Easter Island (known by inhabitants as Rapa Nui). Sirolimus has been shown to be a strong inducer of HbF in *in vitro* systems,^28–31^ *in vivo* animal model systems,^32–34^ and some patients affected by sickle-cell disease (SCD). Currently, this drug is used as an immunosuppressant in combination with other drugs (e.g. cyclosporine or corticosteroids) to prevent transplant rejection^35,36^ (see Supplementary Figure S1 for a historical summary of key actions and findings concerning sirolimus).

The relevance of these reports is due to the fact that no treatment has so far been approved for HbF inducers in thalassemia. Although hydroxyurea (HU) is frequently used (despite the lack of formal approval), its use is limited by its potential adverse effects and reported efficacy in only a subset of patients.^37–39^ Thus, a substantial percentage of patients with thalassemia is not treated with HU and may benefit from other treatments. Moreover, some patients become HU-resistant after long-term treatment.^40^

The working hypothesis to propose sirolimus for the treatment of β-thalassemia is that a sirolimus-dependent increase in HbF levels might improve the clinical status of patients. The aim of this approach is to propose a strategy to reduce the need for transfusion in transfusion-dependent (TDT) patients, who constitute the majority in Europe. For transfusion-independent (NTDT) patients, sirolimus effects should be studied with end-points that are partly different from those described in this article. For instance, ineffective erythropoiesis and peripheral hemolysis are the important factors that cause a variety of subsequent pathophysiological alterations in NTDT, including iron overload and hypercoagulability, which are associated with serious clinical morbidities.^41^ The correction of these factors (for instance, the correction of the α-globin/β-globin imbalance and dysregulated levels of hepcidin) should be considered in clinical protocols for NTDT.^42–44^

Based on published information about the effects of sirolimus on HbF, the orphan drug designation (ODD) for this repurposed drug was obtained by Rare Partners from the European Medicines Agency, for the treatment of β-thalassemia (code: EU/3/15/1585) and SCD (code: EU/3/17/1970). The ODD of sirolimus as an HbF inducer in β-thalassemia and SCD were also obtained from the US Food and Drug Administration (Supplementary Figure S1).

In this article, we report the major biochemical, molecular, and clinical outcomes of the NCT03877809 pilot clinical trial (a personalized medicine approach for β-thalassemia transfusion-dependent patients: testing sirolimus in a first pilot clinical trial: Sirthalaclin), which focused on patients with β^+^thalassemia genotypes.^45^ This trial was based on the use of low dosages of the repurposed drug sirolimus for a 12-month period.^46,47^

The main objective of this interventional, pilot, open-label phase II study with sirolimus in patients with β-TDT (transfusion-dependent thalassemia) was to verify its efficacy as an *in vivo* HbF inducer, with the aim of characterizing a compound with possible application for the reduction of transfusion needs, with an overall good tolerability. The second objective was to determine the possible occurrence of adverse events (AEs) during the clinical trial, resulting from any abnormal laboratory or instrumental findings, symptoms, or disease temporally associated with the use of the investigational product. This pilot study aimed to verify the feasibility of this approach and the need for more complete future trials based on sirolimus.

## Methods

### Patient recruitment and treatment with sirolimus

Recruitment of the Sirthalaclin pilot clinical trial and data collection (EudraCT no: 2018-001942-33, NCT 03877809) was carried out at the Thalassemia Center of Azienda Ospedaliera-Universitaria S. Anna (Ferrara, Italy). The β-thalassemia patients recruited in this study were from patients with the β^+^/β^+^ and β^+^/β^0^ genotypes. The study was approved by the Ethical Committee in charge of human studies at Arcispedale S. Anna, Ferrara (release of approval: 14 November 2018). Patients who fulfilled the inclusion/exclusion selection criteria (listed in Supplementary Tables S1 and S2) received a patient information sheet to read and time to clarify their doubts with the investigators before consenting. Written informed consent was obtained from all participants before they were recruited for the study. The reporting of this study conformed to the Equator Network Guidelines, following the CONSORT pilot and feasibility trial statement.^48^ Adherence to the relevant guidelines is summarized in Supplementary Table S3, which reports the CONSORT checklist of information included in this pilot trial.

**Figure 1.**
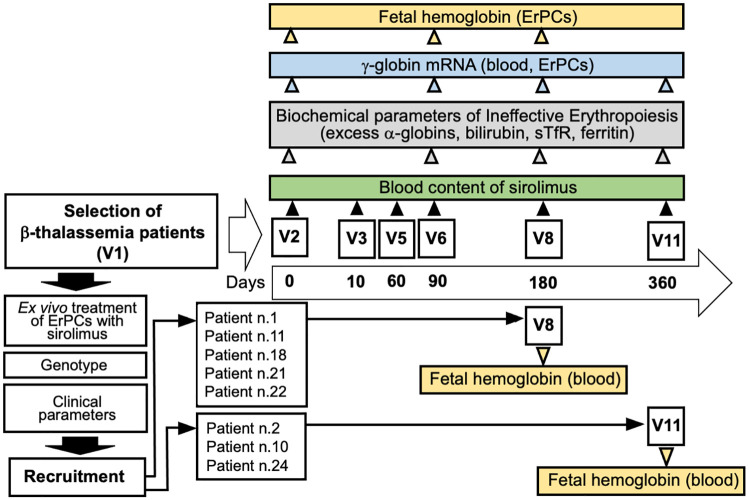
Flowchart summarizing the NCT03877809 Sirthalaclin clinical trial and the key analysis presented in this study. Five patients concluded the trial at V8 (180 days), as indicated. HbF and excess of free α-globin chains were analyzed using HPLC. Content of γ-globin mRNA was analyzed using RT-qPCR. The scheme of the trial has been described elsewhere.^46^

The investigational drug was provided to the patients in the form of coated tablets (0.5 mg sirolimus) in blisters that were adequately labeled with the information regarding the study. At visit 2 (V2, day 0, Figure 1), the patients were instructed to take two tablets (0.5 mg each). The sirolimus level in the blood was measured for the first time after 10 days of therapy and then again on day 90 (visit 6, V6), day 180 (visit 8, V8), day 240 (visit 9, V9), and day 360 (visit 11, V11). The starting sirolimus dose was 1 mg/day. The treatment dose was planned to be increased to 2 mg/day or decreased to 0.5 mg/day to maintain whole blood sirolimus concentration in the range of 5–8 ng/mL. The dosage was maintained constant unless the reduction was dictated by side effects. All other standard treatments, including blood transfusions and iron chelation therapy, were continued. All patients received leuco-depleted packed red blood cell transfusions to maintain a pre-transfusion level of Hb between 9 and 10.5 g/dL, in agreement with the guidelines of the International Thalassemia Federation.^49^

### Analysis of the sirolimus blood content

Analysis of the sirolimus blood content in thalassemia patients was carried out using solid-phase extraction (SPE). The method has been fully described by Ivanova *et al.*^50^ and Navarrete *et al*.,^51^ and is routinely used, with some modifications, to determine sirolimus levels in patients.^52^ Briefly, 100 µL of EDTA-treated whole blood sample/calibrator/control is added to 200 µL of deproteinizing solution [zinc sulfate heptahydrate (0.07 M) in methanol: water 80:20 v/v, VWR West Chester, PA, USA] containing Everolimus-d4 (4 µg/mL) (Sigma-Aldrich, St Louis, MO, USA) as an internal standard and vortexed for 10 s. After centrifugation at 13,000 r/min (16,600*g*) for 5 min, supernatants are transferred to dedicated microplates to be housed in the high-performance liquid chromatography (HPLC) autosampler. The liquid chromatography – mass spectrometry (LC/MS) system consists of a Series 200 Perkin-Elmer HPLC instrument (Perkin-Elmer Italia S.p.A., Milan, IT) coupled with an Ab-Sciex API 2000 Mass Spectrometer (AB Sciex LLC, Redwood City, CA, USA). Injected samples (15 µL) go through an online cleaning step by means of a POROS™ R1/20 preparative column (Thermo Fisher Scientific Inc., Waltham, MA, USA) and are subsequently separated on a Luna Phenyl-Hexyl 50 × 2.1 mm, 5 μm analytical column (Phenomenex Inc., Torrance, CA, USA). Analyses are conducted at 60°C, with a duration of about 3.2 min at a flow of 0.4 mL/min. Mobile phases A (methanol: water 50:50 v/v, MS grade) and B (methanol: water 97:3 v/v containing 10 mM ammonium acetate and 0.1% acetic acid, VWR West Chester, PA, USA) flow according to the following program: from 0 to 1 min, POROS column is charged with 0% of mobile phase B; from 1 to 1.01, a climb up to 100% of phase B; then 100% of phase B is maintained up to 2.8 min. The analysis ends at 3.2 min by rebalancing the system with 0% of phase B. For the mass acquisition, the instrument uses an ionization mode by electrospray ionization (ESI) in a positive polarity, the electrode voltage is set at 5500 V, and the capillary temperature is set at 275°C. The mass acquisition mode is Multiple Reaction Monitoring (MRM), identifying as characteristic transitions for the Sirolimus and Everolimus-d4, respectively, 931.6/864.6 and 981.6/914.4. Seven-level calibrators (Chromsystem GmbH, Munich, Germany) covering a concentration range from 2.4 to 47.0 µg/L and four-level quality controls (Chromsystem GmbH, Munich, Germany) covering a range from 3.0 to 35.0 µg/L are included in each analytical session. This method allows to detect blood sirolimus with a limit of quantitation (LOQ) of 1.0 µg/L and is routinely used by the ‘Laboratorio Unico Metropolitano, Ospedale Maggiore, Azienda USL di Bologna, Bologna, Italy’ for immunosuppressant therapeutic drug monitoring (TDM) in post-transplant patients. Laboratory performance is periodically monitored through proficiency testing participation (External Quality Assessment).

### *In vitro* culture of erythroid precursors from β-thalassemia patients

The two-phase liquid culture procedure was performed as previously described.^29,53,54^ Mononuclear cells were isolated from peripheral blood samples of β-thalassemia patients using Ficoll^®^-Hypaque density gradient centrifugation. After isolation, the mononuclear cell layer was washed three times with 1x phosphate-buffered saline (PBS) solution and seeded in α-minimal essential medium (α-MEM, Sigma-Aldrich, St. Louis, MO, USA) supplemented with 10% fetal bovine serum (FBS; Biowest, Nuaille, France), 1 µg/mL cyclosporine A (Sigma-Aldrich), 10% conditioned medium from the 5637 bladder carcinoma cell line culture, and stem cell factor (SCF; Life Technologies, Monza, MB, Italy) at a final concentration of 10 ng/mL. The cultures were incubated at 37°C, in an atmosphere containing 5% CO_2_. After 7 days in this phase I culture (at 37°C, in an atmosphere containing 5% CO_2_, with extra humidity), the non-adherent cells were harvested from the flask, washed in 1x PBS, and then cultured in phase II medium, composed of α-MEM medium, 30% FBS (Celbio), 1% bovine serum albumin (BSA; Sigma-Aldrich),10^-5^ M β-mercaptoethanol (Sigma-Aldrich), 2 mM L-glutamine (Euroclone, Pero, Milano, Italy),10^-6^ M dexamethasone (Dexamethasone 21-phosphate disodium salt; Sigma-Aldrich), 1 U/mL human recombinant erythropoietin (EPO; Tebu-bio, Magenta, Milano, Italy), and SCF (Life Technologies) at a final concentration of 10 ng/mL. Culture of erythroid precursor cell (ErPC) differentiation was assessed by means of benzidine staining.^29,45,46^ Sirolimus was administered to the ErPCs at a concentration of 100 nM. The yield in terms of percentage of ErPCs was always higher than 85%, following immunological fluorescence-activated cell sorter (FACS) characterization using antibodies recognizing CD71 and CD235a, in agreement with previously reported data.^55^ Representative data are shown in Supplementary Figures S2 and S3.

### RNA extraction from blood and ErPCs

Total cellular RNA was extracted from the blood and ErPCs using TRIzol™ LS (Life Technologies, Monza, MB, Italy) and TRI Reagent^®^ (Sigma-Aldrich, St. Louis, MO, USA), respectively, as per the manufacturer’s instructions. The protocol used for the extraction of whole blood RNA was as follows: (a) 100 µL of whole blood was diluted in three volumes of 1× PBS and processed using 1 mL of TRIzol LS (added without reverse pipetting to avoid any clumping of blood); (b) the isolated RNA was washed once with cold 75% ethanol, dried, and then dissolved in 10–20 µL nuclease-free water before use. The procedure for RNA extraction from ErPCs was similar to that described for the blood. In this case, 800 µL of TRI Reagent was added to a dry pellet consisting of 4–6 × 10^6^ cells.

### Reverse-transcription quantitative PCR analysis of γ-globin gene expression

For gene expression analysis, 500 ng of total RNA was reverse-transcribed using TaqMan^®^ reverse-transcription reagents and random hexamers (Applied Biosystems, Life Technologies, Carlsbad, CA, USA). To quantify the expression of the globin genes, quantitative real-time PCR assay was carried out using two different reaction mixtures – the first containing γ-globin probe and primers, and the second containing GAPDH, RPL13A, β-actin probes, and primers. The primers and probes that were used are listed in Table 1.

**Table 1.** Sequences of the primers and probes employed in this study.

Primer/probe	Sequence
γ-globin forward (primer)	5′-TGACAAGCTGCATGTGGATC-3′
γ-globin reverse (primer)	5′-TTCTTTGCCGAAATGGATTGC-3′
γ-globin probe	5′-FAM-TCACCAGCACATTTCCCAGGAGC-BFQ-3′
RPL13A forward (primer)	5′-GGCAATTTCTACAGAAACAAGTTG-3′
RPL13A reverse (primer)	5′-GTTTTGTGGGGCAGCATCC-3′
RPL13A probe	5′-HEX-CGCACGGTCCGCCAGAAGAT-BFQ-3′
β-actin forward (primer)	5′-ACAGAGCCTCGCCTTTG-3′
β-actin reverse (primer)	5′-ACGATGGAGGGGAAGACG-3′
β-actin probe	5′-Cy5-CCTTGCACATGCCGGAGCC-BRQ-3′
GAPDH forward (primer)	5′-ACATCGCTCAGACACCATG-3′
GAPDH reverse (primer)	5′-TGTAGTTGAGGTCAATGAAGGG-3′
GAPDH probe	5′-FAM-AAGGTCGGAGTCAACGGATTTGGTC-BFQ-3′

Each reaction mixture contained 1x Ex Taq™ DNA Polymerase (Takara Bio, Shiga, Japan), 500 nM forward and reverse primers, and 250 nM probes (Integrated DNA Technologies, Castenaso, Italy). Assays were carried out using the CFX96™ Touch Real-Time PCR System (Bio-Rad, Hercules, CA, USA). After initial denaturation at 95°C for 1 min, the reactions were performed for 50 cycles (95°C for 15 s and 60°C for 60 s). Data were analyzed using CFX Manager™ software (Bio-Rad). The ΔΔCt method was used to compare the gene expression of each amplified template.^29,30^

### HPLC analysis of Hbs

To evaluate the effectiveness of the treatment, HPLC analysis was performed using both blood and ErPCs. ErPCs were centrifuged at 1200 r/min for 6 min and washed with PBS. The pellets were re-suspended in a pre-defined volume of water for HPLC (Sigma-Aldrich). This was followed by three freeze/thaw cycles on dry ice to lyse the cells and obtain protein extracts. Lysates were centrifuged for 5 min at 14,000 r/min, following which the supernatant was collected. Hb analysis was performed by loading the protein extracts into a PolyCAT-A cation exchange column and then eluting in a sodium-chloride-BisTris-KCN aqueous mobile phase using an HPLC Beckman Coulter System Gold 126 Solvent Module-166 Detector (Beckman Coulter Inc, Brea, CA, USA), which allowed us to quantify the Hb present in the sample. The reading was performed at a wavelength of 415 nm, with a commercial solution of purified human HbAF (Analytical Control Systems Inc., Fishers, IN, USA) extract used as the standard. The obtained values were processed using 32 Karat software (Beckman Coulter).

### Calculation of transfusion indices

Transfusion episodes occurred in two periods: prior to sirolimus treatment (approximately 180 days before V2) and during sirolimus treatment (from V2 to V8). For each period, transfusion indices, including the average pre-transfusion Hb concentration (g/dL) and red cell consumption (ml of pure red cells per kg body weight per year), were calculated. The index of transfusion demand was calculated by dividing red cell consumption by the average pre-transfusion Hb concentration. If the endogenous production of Hb increased on sirolimus treatment, this parameter was expected to decrease proportionally. Details about and a representative example of the calculation of the transfusion indices are included in Supplementary Materials.

Erythroblast, reticulocyte, total bilirubin, lactate dehydrogenase (LDH), soluble transferrin receptor (sTFR), EPO, and ferritin levels were determined using standard clinical assays at the Laboratory of Analysis, Arcispedale S. Anna di Ferrara, Ferrara, Italy.

### Analysis of the immunophenotype

For the immunophenotype analysis performed using flow cytometry, peripheral blood mononuclear cells (PBMCs) were isolated from whole blood of recruited patients during different time-points (V2, V6, V8, and V11). Briefly, a small amount of blood was layered on Ficoll and centrifuged to collect the buffy coat (mainly white blood cells, ErPCs, and platelets), followed by a series of washes in PBS to clean the cells and eliminate the platelet component. At the end of the process, the isolated PBMCs were frozen in several cryovials using FBS with 10% dimethyl sulfoxide as a cell cryopreservation medium and stored in liquid nitrogen. At the end of the clinical trial, for each patient, we thawed one cryovial for each time-point to perform the immunophenotype analysis. Cells were thawed, centrifuged in Roswell Park Memorial Institute (RPMI) medium, washed in PBS, and counted; one million cells were selected for each time-point to proceed with antibody staining. The cells were stained with the LIVE/DEAD™ Fixable Aqua Dead Cell Stain Kit (Thermo Fisher, Waltham, MA, USA) and incubated at 10°C in the dark to stain cells with lost membrane integrity. After washing with PBS, the cells were stained with a mixture of antibodies (Table 2) that target different membrane receptors (antibodies against CD3, CD4, CD8, CD14, CD19, and CD25 markers) and incubated for 15 min in the dark. At the end of the staining process, the cells were washed with PBS to reduce the background, re-suspended in 200 µL of PBS, and analyzed by means of flow cytometry using a BD FACSCanto™ II cell analyzer (Becton Dickinson, Franklin Lakes, NJ, USA).^56–58^

**Table 2.** Antibodies used for immunophenotype analysis of PBMCs.

Ab	Fluorochrome	Catalog no.	Company
CD19	PB	302232	BioLegend (San Diego, CA, USA)
CD25	PE	12-0259-42	Invitrogen, Thermo Fisher, Waltham, MA, USA
CD3	PerCP	300428	BioLegend
CD14	PE-Cy7	25-0149-42	Invitrogen, Thermo Fisher
CD4	APC	300514	BioLegend
CD8	APC-Cy7	557834	BD PharMingen, Franklin Lakes, NJ, USA

### Statistical analysis

All data were normally distributed and have been presented as mean ± standard deviation (SD). Statistical differences between groups were compared using paired *t*-test or one-way repeated measures analysis of variance, followed by least significant difference (LSD) *post hoc* test. Statistical differences were considered significant when *p* < 0.05 (*) and highly significant when *p* < 0.01 (**).

## Results

### Selection of β-thalassemia patients and determination of the *in vitro* response of ErPCs to sirolimus

A patient information sheet was provided to all patients who fulfilled the inclusion/exclusion selection criteria (enlisted in Supplementary Tables S1 and S2). Time necessary to discuss and clarify all doubts with the investigators was allowed before consent was obtained. Informed written consent was obtained from all the participants before they were recruited for the study. At the time of enrollment, information on socio-demographic background, family history, medical history, present medical problems, and concomitant medications was reviewed. During the study selection period, physical examination, cardiac evaluation, abdominal ultrasound, and blood tests were performed. Patients who fulfilled the inclusion/exclusion criteria and were responsive to sirolimus *in vitro* (according to laboratory-specific definition: ⩾20% increase in HbF, in comparison to samples not treated with sirolimus) were eligible for the study and were recruited.

Figure 1 shows a pictorial flowchart summarizing the key steps of the NCT03877809 Sirthalaclin clinical trial, including the preliminary phases allowing recruitment of β-thalassemia patients and the scheme of the biochemical and molecular assays conducted and presented in this article.

**Figure 2.**
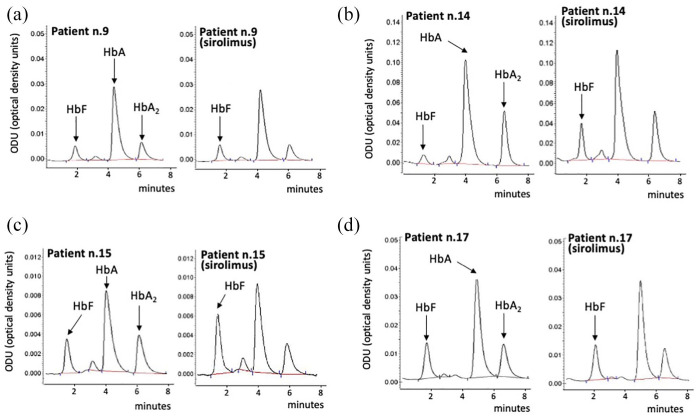
Representative HPLC patterns obtained after exposure of ErPCs isolated from patients (a) n.9, (b) n.14, (c) n.15, and (d) n.17 to sirolimus. The arrows indicate the positions of the HbA, HbA_2_, and HbF_0_ peaks.

As indicated in the previously presented protocol^46^ and in Figure 1, ErPCs from the patients were initially treated with the drug *in vitro*, and only those patients whose cells were responsive *in vitro* were then considered as candidates for oral administration of the drug. A total of 24 β^+^-thalassemia patients were recruited for this analysis. This number was deemed sufficient for the identification of about 10 patients who were able to respond to sirolimus in terms of increased HbF production.^45,46^ Representative HPLC patterns obtained after exposure of ErPC isolated from patients n.9, n.14, n.15, and n.17 to sirolimus are shown in Figure 2. The arrows indicate the positions of the Hb peaks, including the major HbF peak (HbF_0_). The ErPCs cultures from two patients (n.9 and n.17) were not responders (increase in HbF < 20%), while the ErPC cultures from two other patients (n.14 and n.15) were considered responders (increase in HbF > 20%). The data obtained from the ErPCs of the 24 patients studied during the selection period are reported in Table 3. A total of 16 (66.6%) patients were found to have ErPCs that were responsive to the sirolimus treatment, as stated in the Sirthalaclin protocol (i.e. 20% increase after *in vitro* induction of the ErPCs with sirolimus).

**Table 3.** *In vitro* response of ErPCs to sirolimus.

Patient no. (*)	Baseline HbF (**)	*In vitro* induction (% increase)	Recruitable	Treatment with sirolimus
01	17.80	21.72	Yes	Yes
02	10.31	47.40	Yes	Yes
03	18.15	29.51	Yes	No
04	45.09	11.31	No	n.a.
05	33.11	14.72	No	n.a.
06	17.70	20.28	Yes	No
07	9.06	44.92	Yes	No
08	17.33	21.20	Yes	No
09	12.07	16.30	No	n.a.
10	14.91	28.23	Yes	Yes
11	15.02	24.82	Yes	Yes
12	11.49	22.37	Yes	No
13	8.89	14.80	No	n.a.
14	8.32	61.00	Yes	No
15	16.74	61.91	Yes	No
16	11.10	16.91	No	n.a.
17	18.38	8.31	No	n.a.
18	21.32	29.11	Yes	Yes
19	33.18	10.50	No	n.a.
20	7.52	19.50	No	n.a.
21	9.47	24.71	Yes	Yes
22	11.24	29.70	Yes	Yes
23	19.27	35.11	Yes	No
24	14.95	26.31	Yes	Yes

n. a., not applicable.

(*) Patients were randomized to avoid identifying information.

(**) Analyzed using HPLC and expressed as percentage of total Hb.

Among them, eight patients decided to start sirolimus intake (Table 3**)**. The age of these patients was between 40 and 60 years (four were in the range of 40–45 years, while two were in the range of 46–50 years, and two were in the range of 56–60 years). The most represented genotype was β^0^39/β^+^IVSI-110 (found in six patients). One patient had β^+^IVSI-110/β^+^IVSI-110 genotype, while another had β^0^39/β^+^IVSI-6 genotype. The female-to-male ratio was 6:2. Most patients (six patients) underwent splenectomy. Other clinical and biochemical parameters are listed in Supplementary Table S4.

### Sirolimus dosage in blood

The starting sirolimus dosage was 1 mg/day, with planned minimum and maximal doses of 0.5 and 2.0 mg/day, respectively. The dosage was adjusted based on blood levels and tolerability. The analysis of sirolimus levels in the peripheral blood is shown in Table 4. We observed high variability among patients and relatively low levels of blood sirolimus concentrations in all sirolimus-treated patients. Note that at 90 and 180 days, eight patients were taking sirolimus (average dose 1.2 and 1.7 mg/day, respectively). Later, only three patients remained under treatment, and their blood levels were not substantially different.

**Table 4.** Levels of sirolimus in the blood (ng/mL).

Patient no.	V2	V3	V5	V6	V8	V9	V11
1	n.d. (*)	2.7	1.1	2.0	<1.0	(**)	(**)
2	n.d.	2.9	3.7	2.1	2.0	5.1	n.a.
10	n.d.	3.1	2.7	4.5	2.4	5.9	4.4
11	n.d.	2.0	3.3	1.9	3.7	(**)	(**)
18	n.d.	4.2	3.6	2.7	4.6	(**)	(**)
21	n.d.	2.1	<1.0	1.7	3.0	(**)	(**)
22	n.d.	1.6	1.1	2.4	<1.0	(**)	(**)
24	n.d.	2.9	2.1	1.5	n.a.(*)	4.6	n.a.

(*) n.d., not detected; n.a., not available.

(**) Patients 1, 11, 18, 21, and 22 concluded the trial at V8.

**Figure 3.**
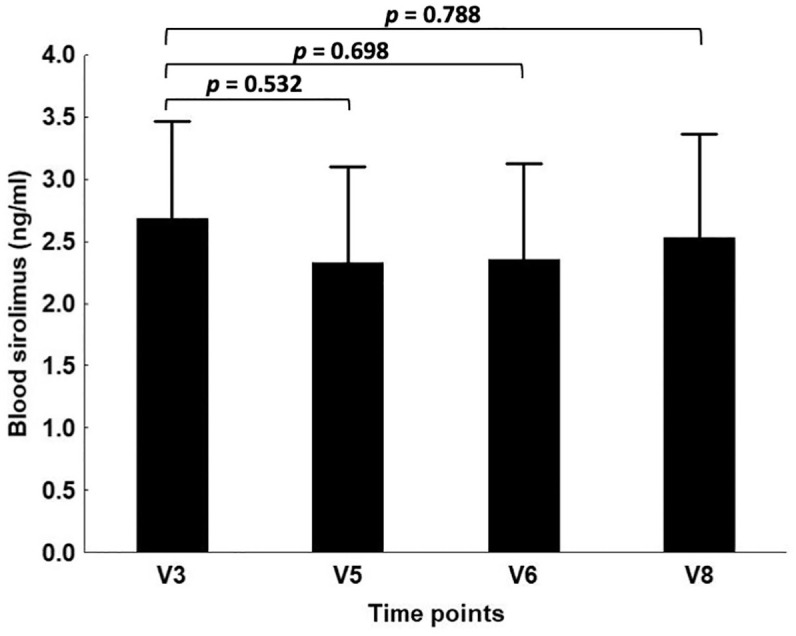
Blood levels of sirolimus (ng/mL). For calculating the average values, the samples with sirolimus blood concentrations <1.0 ng/mL were arbitrarily considered as equal to 1.0 ng/mL. In the calculation of *p*-value for the comparison of V8 data to V3 data, samples from patient n.24 were not considered.

To compare blood levels at different time-points, we considered that samples with a sirolimus concentration close to the detection limit of 1 ng/mL as containing 1 ng/mL sirolimus. Using this procedure, the average values at V3, V5, V6, and V8 (Figure 3) were not different according to the analysis of variance test (*p* = 0.934; Figure 3), indicating that repeated administration of low-dose sirolimus did not cause accumulation of the drug.

### Evidence for increased γ-globin mRNA content in the blood

To quantify the γ-globin mRNA content in the blood of sirolimus-treated patients, we developed a reverse-transcription quantitative PCR (RT-qPCR) technique to amplify mRNA sequences of γ-globin and RPL13A, GAPDH, and β-actin control sequences from the peripheral blood. This technique allowed us to highlight an increase in γ-globin mRNA levels in the blood of most patients treated with sirolimus.

**Figure 4.**
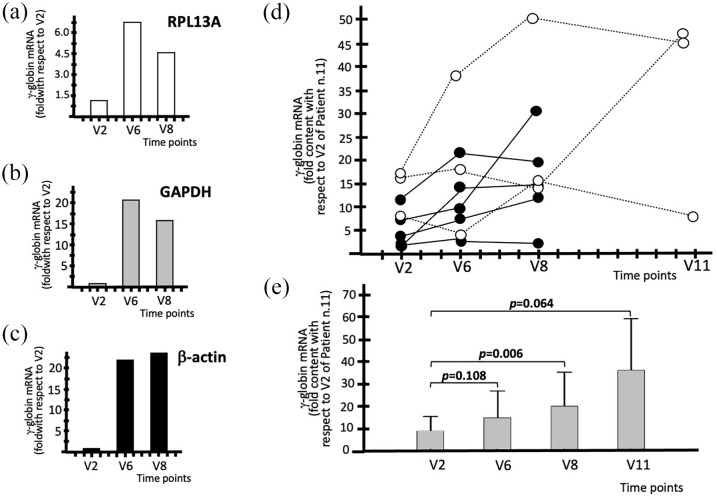
Increase in the content of γ-globin mRNA in the blood of patients treated with sirolimus (a–c). Representative data obtained from the blood samples of patient n.11 using (a) RPL13A, (b) GAPDH, and (c) β-actin control sequences, as indicated. (d) Increase in the content of γ-globin mRNA in the blood. The data represent fold values with respect to V2 of patient n.11. (e) Average increase values of γ-globin mRNA expressed as fold content with respect to V2 of patient n.11. Raw data are presented in Supplementary Table S5. *N* = 8 (V2–V8) and 3 (V11).

The experiment illustrated in Figure 4(a)–(c) demonstrates a clear increase in γ-globin mRNA in the blood of a patient treated with sirolimus and that this was not affected by the choice of the internal standards. In addition, an increase was evident in V6. Moreover, there was a selective increase in the blood content, as evidenced by comparing the RT-qPCR results specific for α-globin, β-globin, and γ-globin mRNAs. The results are shown in Supplementary Figure S4.

In analyzing the data obtained for all sirolimus-treated patients, we should consider that the patients are highly heterogeneous with respect to endogenous production of γ-globin mRNA. This is an expected result considering the HPLC analyses of the patients recruited in the Sirthalaclin trial, as already reported in Table 3. The percentage of HbF varies from 9.47% (in patient n.21) to 21.32% (in patient n.18).

The key results reported in Figure 4(d) demonstrate that the increase in γ-globin mRNA content is clearly appreciable when the RT-qPCR data are normalized with respect to V2 of patient n.11 (expressing lower amounts of γ-globin mRNA). This calculation is important because it provides information about the baseline levels of γ-globin mRNA in the blood of the analyzed patients. For instance, the blood samples of patient n.10 display a 17.71 relative value of γ-globin mRNA at V2 (with respect to the V2 value of patient n.11). Interestingly, this value increased to 38.78 at V6, 50.83 at V8, and 48.8 at V11. Considering the late Sirthalaclin visits (V8 and V11), all the sirolimus-treated patients exhibited increased γ-globin mRNA content, with the exception of patient n.21. As expected, when the data for each patient were compared with their V2 data, a similar trend was observed. In this case, the results are expressed as fold increases with respect to each V2. For instance, when the patient n.10 was considered, the γ-globin mRNA fold increase was 2.19 at V6, 2.87 at V8, and 2.76 at V11. Figure 4(e) shows the average increase in γ-globin mRNA in V6–V11, when all the data are reported relative to the V2 blood sample of patient n.11 (we performed this data analysis usually using the sample exhibiting the lower amount of γ-globin mRNA, which in this case is patient n.11, as reference). The difference when the V8 values were compared to V2 was statistically significant (*p* = 0.006) when the *post hoc* test was performed. All raw data are presented in Supplementary Table S5.

In conclusion, RT-qPCR analysis using blood samples from sirolimus-treated patients showed that the content of γ-globin mRNA increased in samples from all patients, with the exception of patient n.21.

### Evidence for increased γ-globin mRNA content in ErPCs

ErPCs were isolated from the sirolimus-treated patients at V6, V8, and V11. As in case of the blood samples (Figure 4, panel E and Supplementary Table S5), all the comparisons were made with V2 exhibiting the lower γ-globin mRNA level (in this case, patient n.24).

ErPC cultures were conducted in the presence of EPO, but in the absence of sirolimus, to ensure that the observed differences were caused by *in vivo* sirolimus treatment.

**Figure 5.**
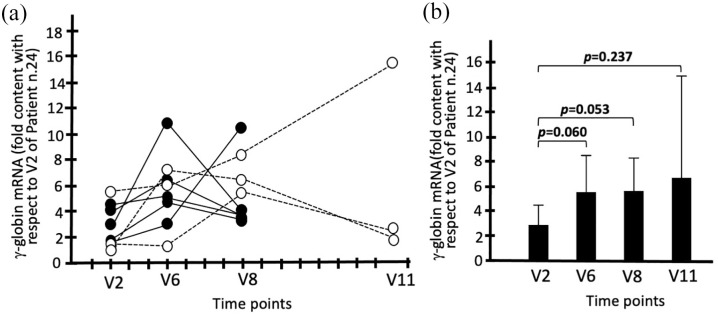
Increase in the content of γ-globin mRNA in the ErPCs isolated from patients treated with sirolimus. (a) Increase in the content of γ-globin mRNA in the ErPCs. The data represent fold values with respect to V2 of patient n.24. (b) Average increase in the content of γ-globin mRNA. The data represent fold values with respect to V2 of patient n.24. Raw data are presented in Supplementary Table S6. *N* = 8 (V2–V8) and 3 (V11).

Supplementary Table S6 and Figure 5 show the data obtained using RT-qPCR, which clearly demonstrates a borderline significant increase in γ-globin mRNA in ErPCs from sirolimus-treated patients. This was observed at both V6 (*p* = 0.060) and V8 (*p* = 0.053). This trend was observed despite the variability in the increase in γ-globin mRNA in V2 samples, which reflect the baseline γ-globin mRNA levels in ErPCs of the different β-thalassemia patients analyzed. All raw data are presented in Supplementary Table S6. It should be considered that sirolimus was absent during EPO treatment of the ErPCs; therefore, all the changes observed between the samples in V2 and those in V6, V8, and V11 should be ascribed to the *in vivo* effects of sirolimus on the studied patients.

In conclusion, RT-qPCR analysis using EPO-cultured ErPC samples from sirolimus-treated patients supports the concept that the expression of γ-globin genes increased in samples from all the patients, with the exception of patients n.1 and n.21, who should be considered low responders. In addition, it should be noted that the increase in sirolimus-induced γ-globin mRNA is already detectable at V6, both when samples of peripheral blood (Figure 4) and ErPCs (Figure 5**)** are considered.

### Increase in HbF levels in ErPCs from sirolimus-treated patients: HPLC analyses

**Figure 6.**
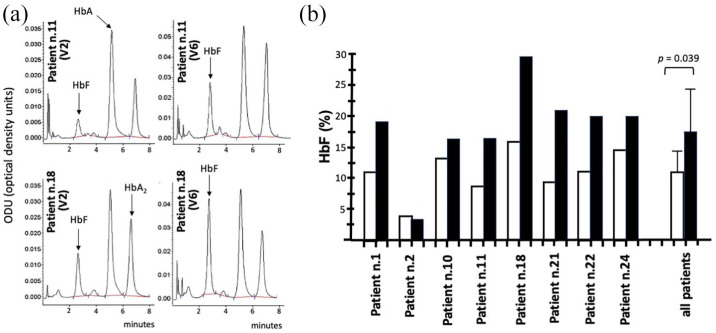
Increase in percentage of HbF in EPO-induced ErPCs isolated at V6. (a) Representative HPLC analysis of EPO-treated ErPCs from patients n.11 and n.18 at V2 and V6 Sirthalaclin visits. The arrows indicate positions of the HbA, HbA_2_, and HbF peaks. (b) Changes in the percentage of HbF at V6 (black histograms) with respect to V2 (white histograms). The cumulative data from all the analyzed patients are also shown.

Figure 6(a) shows representative HPLC patterns of V6 samples obtained by stimulating ErPCs isolated from patients n.11 and n.18 with EPO. In both cases, the percentage of HbF increased in V6 samples, as compared to that in V2 samples. In samples from patient n.18, the percentage of HbF increased from 16.33 (V2) to 29.38 (V6) (1.80-fold), while in samples from patient n.11, the percentage of HbF increased from 8.79 (V2) to 16.41 (V6) (1.88-fold). Data related to EPO-stimulated ErPCs from all patients at V6 are shown in Figure 6(b).

The data obtained, which are fully in agreement with the RT-qPCR data shown in Supplementary Table S6 and Figure 5(a), demonstrate increased HbF production in ErPCs isolated at V6 from all the patients, with the exception of patient n.2. The mean increase in HbF% was 1.57, which was statistically significant (*p* = 0.039).

In conclusion, HPLC analysis using EPO-cultured ErPC samples from sirolimus-treated patients strongly supports the finding that HbF production increased at V6, in full agreement with the RT-qPCR data shown in Figure 5.

### Effects of sirolimus on biomarkers associated with ineffective erythropoiesis

Several biochemical markers are known to be associated with ineffective erythropoiesis and might be important parameters in determining the *in vivo* activity of HbF inducers, including sirolimus.^59–64^ The following parameters were measured before and under sirolimus treatment, as indices of erythroid response: erythroblast count, reticulocytes count and total bilirubin, LDH, sTFR, EPO, and ferritin levels. While no significant difference was observed in erythroblast count, reticulocytes count, LDH, and EPO levels under treatment with sirolimus *versus* baseline (data not shown), a significant reduction was found in total bilirubin, sTFR, and ferritin levels. In addition to these markers, we analyzed the possible inhibitory effects of sirolimus on the excess free α-globin chains produced by ErPCs. In this context, an excess of free α-globin chains has been firmly demonstrated to correlate with red blood toxicity. It is known that all these parameters participate in a complex network that affects erythropoiesis (and ineffective erythropoiesis) and iron metabolism balance.^60–64^

**Figure 7.**
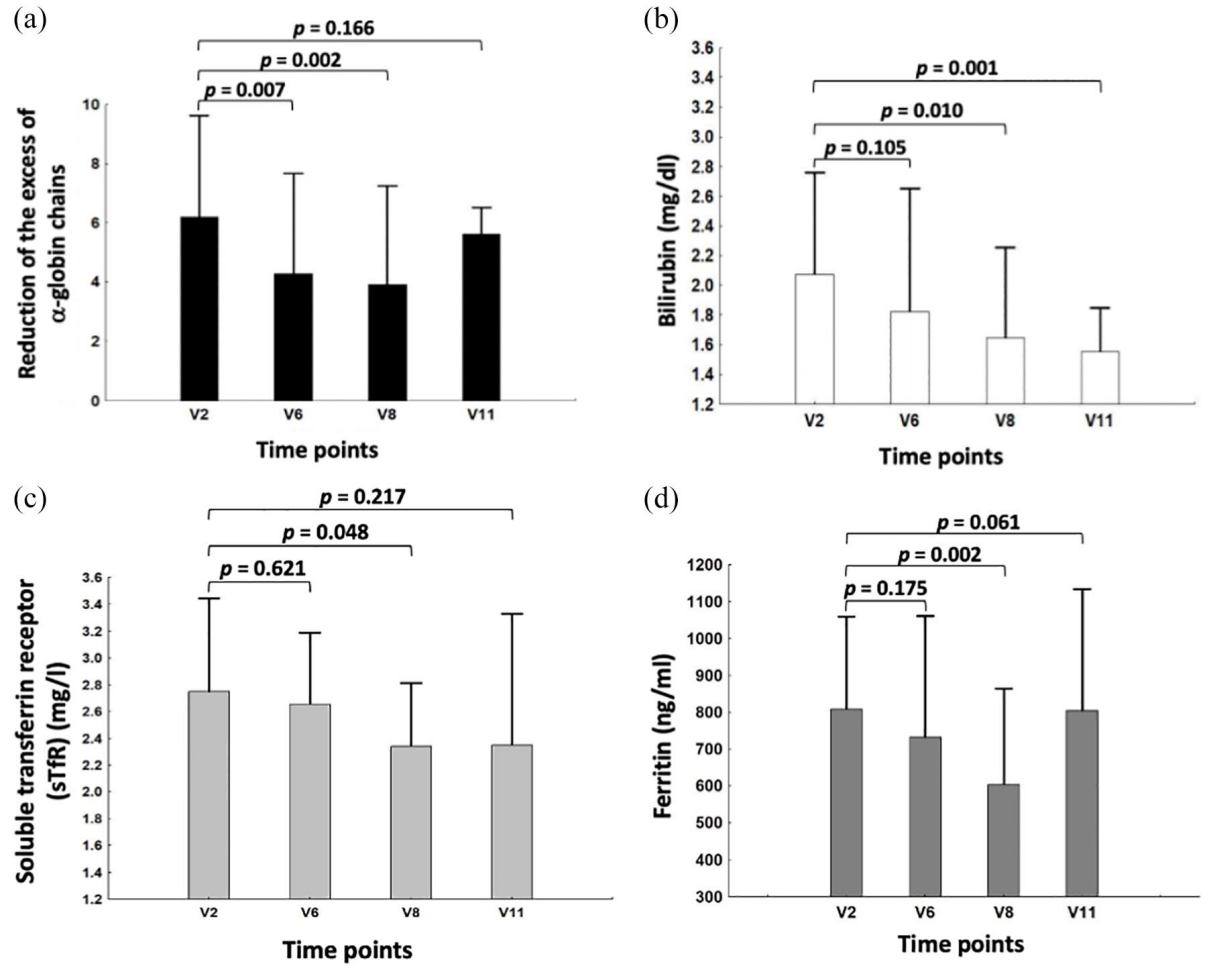
(a) Changes in free α-globin chains, (b) bilirubin, (c) sTFR, and (d) ferritin levels, following treatment with sirolimus. Only six patients were analyzed for changes of free α-globin chains, since two patients exhibited lack of detectable α-globin peak on HPLC analysis. Raw data related to panels B–D are presented in Supplementary Tables S7**–**S9. *N* = 8 (V2–V8) and 3 (V11).

Figure 7 shows the sirolimus-mediated changes in the free α-globin chains, bilirubin, sTFR, and ferritin levels. The data obtained indicate that there is a consistent reduction in free α-globin chains in ErPCs isolated from β-thalassemia patients treated with sirolimus [Figure 7(a)]. This reduction was significant at V6 (*p* = 0.007) and V8 (*p* = 0.002). This is relevant because excess of free α-globin chains is one of the most important causes of red blood cell toxicity.

Moreover, a statistically significant decrease in bilirubin [Figure 7(b)], sTFR [Figure 7(c)], and ferritin [Figure 7(d)] levels was also found at V8. All of these are markers of ineffective erythropoiesis and have been demonstrated to be expressed at high levels in the plasma of β-thalassemia patients. Notably, the significance of these changes was found at V8 and V11 time periods for bilirubin, that is, after the increase in γ-globin and HbF found at V6 (Figures 5 and 6). Overall, these data suggest that sirolimus treatment has a positive effect on ineffective erythropoiesis.

### Evidence for a clinical effect in sirolimus-treated patients at the end of the treatment: indices of transfusion demand and blood HbF

**Figure 8.**
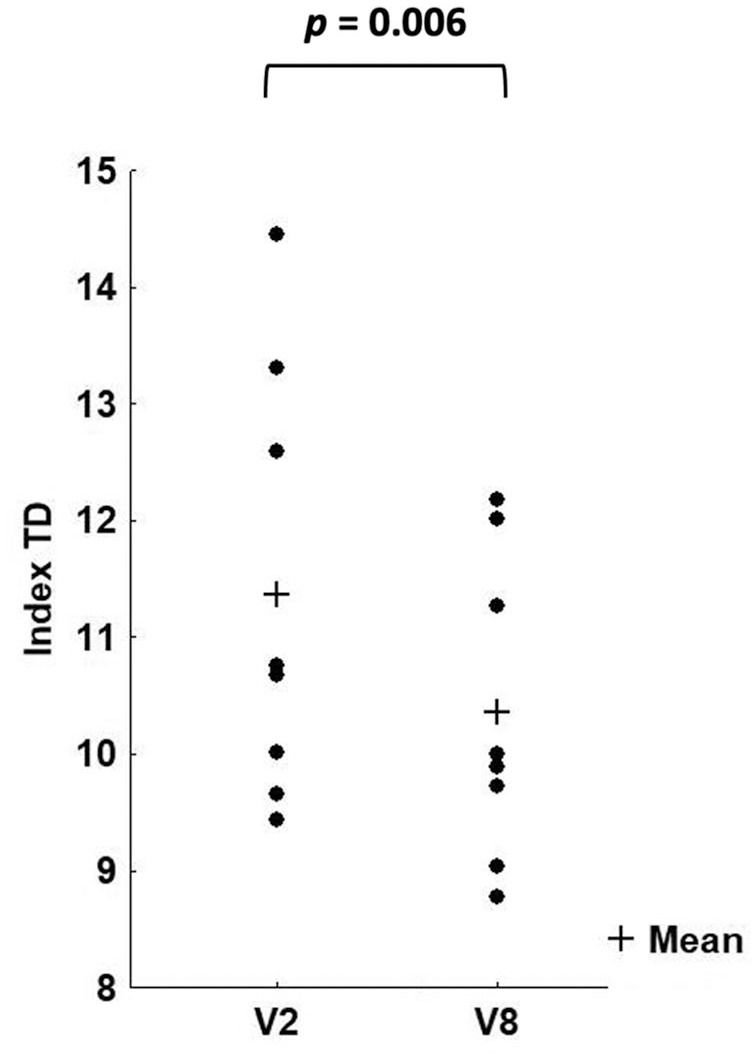
Changes in the transfusion demand (TD) when data at V2 and V8 are considered (*p* = 0.006). *N* = 8.

The index of transfusion demand was calculated by dividing red cell consumption by the average pre-transfusion Hb concentration (see ‘Methods’ section). If the endogenous production of Hb increased under sirolimus treatment, this parameter was expected to decrease proportionally. As shown in Figure 8 and Table 5, a reduction in this parameter was observed. This reduction was statistically significant (*p* = 0.006) (Figure 8).

**Table 5.** Index of transfusion demand (mL/kg/year/gHb).

Patient no.	V2	V8	% change
1	13.31	12.01	−9.77
2	10.76	10.00	−7.04
10	9.66	8.77	−9.28
11	10.02	9.04	−9.79
18	10.68	9.89	−7.37
21	9.43	9.73	3.17
22	14.45	12.18	−15.70
24	12.59	11.27	−10.51

In parallel, an increase in blood HbF was found at the end of the sirolimus treatment in all the patients, with the exception of patients n.21 and n.22 (the percentage change with respect to V2 was −51% and −23%, respectively). The average fold increase in HbF was 1.22 (*SD* = 0.33), which was close to statistical significance (*p* = 0.076). On not considering patient n.21 (who was also not a responder in the transfusion demand assay), the new calculated fold increase was highly significant (*p* < 0.001). The best increase in blood HbF was found in the blood of patients n.1, n.2, and n.18 (+38%, +49%, and +55%, respectively). Intermediate HbF blood increase (+21/+22%) was found in patients n.10, n.11, and n.24.

Notably, the parameter of patient n.21 (increase in transfusion demand and no increase in blood HbF content) was in agreement with the lack of increase in the content of γ-globin mRNA in the blood.

### Immunophenotype before and after sirolimus administration

**Figure 9.**
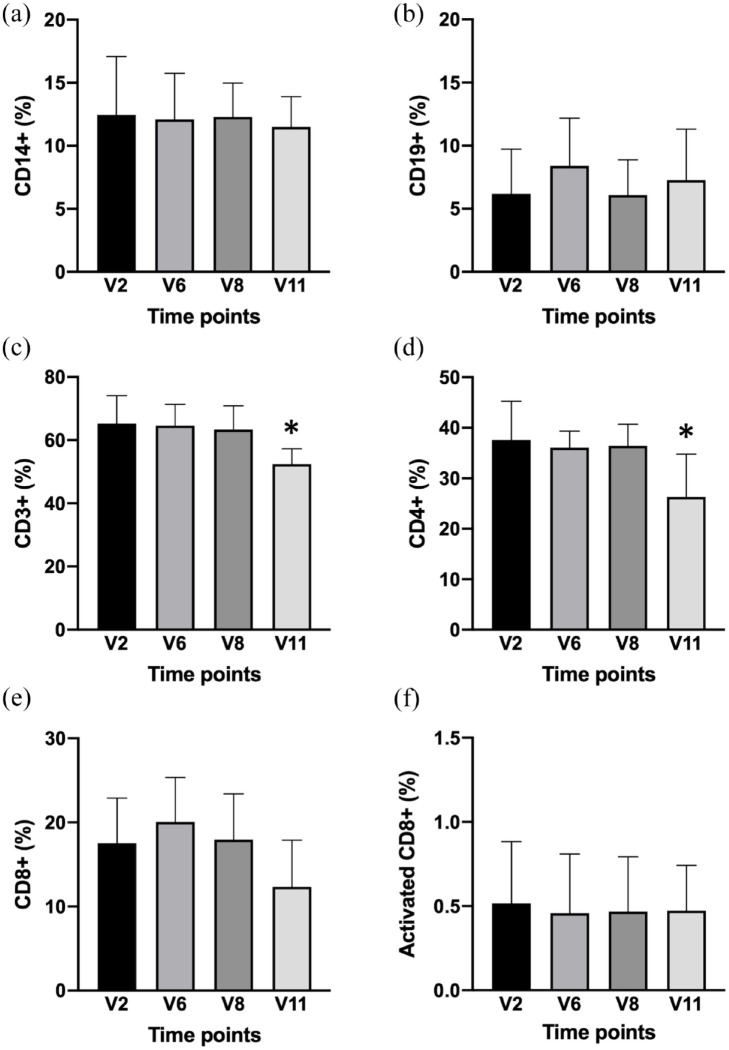
Immunophenotype of PBMCs from sirolimus-treated β-thalassemia patients: FACS analysis. A representative example of the gating strategy employed is given in Supplementary Figure S5A–C. The summary of the results obtained is shown focusing on the following markers associated with the various lymphocyte subpopulations: (a) CD14, (b) CD19, (c) CD3, (d) CD4, (e) CD8, and (f) CD25. With this panel of antibodies, we were able to subdivide the PBMCs population into monocytes (CD14^+^), B cells (CD14^−^/CD3^−^/CD19^+^), T cells (CD14^−^/CD3^+^), CD4^+^ T cells (CD14^−^/CD3^+^/CD4^+^), CD8^+^ T cells (CD14^−^/CD3^+^/CD8^+^), and CD8^+^ activated T cells (CD14^−^/CD3^+^/CD8^+^/CD25^+^). Average changes of the immunophenotype pattern at V2, V6, V8, and V11 are reported. *N* = 8 (V2–V8) and 3 (V11).

As shown in Figure 9, the patients did not show significant changes in the patterns of lymphocyte parameters. The analysis was performed using flow cytometric techniques to assess the following markers associated with the various lymphocyte subpopulations: CD3, CD4, CD8, CD14, CD19, and CD25. The starting material for the analysis consisted of PBMCs obtained from the peripheral blood of the patients. The PBMCs extracted from the blood were frozen and stored in liquid nitrogen until the time of analysis. Before proceeding with incubation with specific antibodies to detect the aforementioned markers, Aqua Dead Cell Stain, which is capable of discriminating between live and dead cells, was used to accurately identify only the intact cells, after defrosting. As an example of the same, the data obtained on analyzing the cells from patient n.18 using the FACS apparatus are shown in Supplementary Figure S5.

As shown in Figure 9, the analyzed patients did not undergo significant variations in the percentages of the various cellular subsets obtained using immunophenotyping (*p* > 0.05), with the exception of the CD3^+^ and CD4^+^ samples of V11, which were significantly lower (*p* < 0.031 and *p* < 0.022, respectively). Therefore, we can conclude that even after 12 months of chronic therapy with sirolimus, thalassemia patients showed only minor alterations in the profile of lymphocyte typing. The recorded values were expressed as a percentage of positive cells for a given marker, with respect to the total number of live cells analyzed, with the exception of effector T lymphocytes (activated CD8^+^), which were expressed as a percentage of CD8^+^ lymphocytes.

### AEs

AEs were classified as expected or unexpected based on their severity (serious, mild, moderate, or severe), according to the Common Terminology Criteria for AEs (version 5.0). They were also considered to be related to investigational drugs. No major AEs were found in the treated β-thalassemia patients. However, despite detailed instructions given to each patient to prevent and treat oral lesions early, 11 episodes of stomatitis [consistent with minor side effects associated with the activity of mammalian target of rapamycin (mTOR) inhibitors]^65–68^ were observed in five patients, and further classified as mild or moderate. Three patients independently decided to cease the trial, owing to the recurrence of stomatitis. A detailed analysis of the observed stomatitis is presented in Supplementary Table S10.

## Discussion

Sirthalaclin was the first pilot clinical trial for β-thalassemia based on the use of sirolimus.^45–47^ This open-label monocentric study was planned to enroll approximately 30 transfusion-dependent patients with β^+^/β^+^ and β^+^/β^0^ thalassemia genotypes and treat their ErPCs with sirolimus to identify an expected number of 20 patients whose ErPCs were responsive to sirolimus *in vitro* (level of HbF, sirolimus concentration 100 nanomolar). Total enrollment (on signature of the informed consent) was for 24 patients, and *in vitro* response was found in 17 of those patients; however, of these, only 8 decided to take sirolimus by the oral route of administration (0.5–2.0 mg/day). None of the patients had to discontinue sirolimus for safety reasons. Out of the eight patients, we have data for all of them at 6 months and for three of them at 12 months.

The main purpose of this study was to determine whether treatment with sirolimus induces an increase in the expression of γ-globin genes in β-thalassemia patients. In this case, a more comprehensive trial should be considered in the future. Several studies that supported this trial were published. First, sirolimus was found to induce HbF accumulation in ErPCs isolated from β-thalassemia and SCD patients.^28–31^ Second, *in vivo* experiments using animal systems demonstrated that sirolimus treatment increases red blood cell counts and Hb levels, thus supporting the hypothesis that sirolimus administration may be beneficial in patients with ineffective erythropoiesis.^59^ In addition, sirolimus was found to induce γ-globin genes, accumulate HbF, and improve anemia in model systems of SCD.^33^ Sirolimus treatment prolonged the lifespan of sickle-cell erythrocytes in circulation, reduced the spleen size, and reduced renal and hepatic iron accumulation in SCD mice.^34^ Third, sirolimus or sirolimus analogs (either alone or in combination with hydroxycarbamide) induced HbF production in patients treated with kidney transplantation.^35,36^ These studies demonstrated that in SCD patients treated with sirolimus or sirolimus analogs, there was a dramatic increase in the HbF level.

In addition to its effects on γ-globin genes and HbF production, sirolimus has been shown to retain other biological activities that are expected to be useful in the treatment of hemoglobinopathies, including β-thalassemia. In a mouse model of thalassemia, Lechauve *et al.*^59^ found that treatment with sirolimus was associated with a significant reduction in α-globin accumulation and ineffective erythropoiesis, along with a longer life span of red blood cells, a combination of effects that is potentially important for patients. Another possible *in vivo* effect of sirolimus might be the enhancement of hematopoietic stem cells through the regulation of the mTOR pathway.^69^ This interesting possibility is supported by the findings suggesting that sirolimus, together with other biological response modifiers, participates *in vivo* in increasing the number of long-term hematopoietic stem cells.^64^

Information on this study (focusing on β^+^/β^+^ and β^+^/β^0^ thalassemia genotypes) can be found at NCT03877809^45^ and in the review by Gamberini *et al.*^46^ In addition, a second study is ongoing (NCT04247750), focusing on β^0^/β^0^ and β^+^/β^0^ thalassemia genotypes.^46^

The first finding of this study is an increased expression of γ-globin mRNA in the blood and ErPCs isolated from β-thalassemia patients treated with low-dose sirolimus. This effect was observed early during the trial (i.e. after 90 days of treatment, at the V6 visit). This finding was fully in agreement with HbF accumulation in ErPCs from sirolimus-treated β-thalassemia patients. An increase in HbF level was observed when HPLC was performed at V6. It should be emphasized that ErPCs isolated from sirolimus-treated β-thalassemia patients were cultured with only SCF and EPO (without sirolimus), suggesting that the changes in gene expression found should be ascribed to *in vivo* treatment with sirolimus. This is the first description of an increase in γ-globin mRNA expression in β-thalassemia patients treated with low-dose sirolimus.

The interest in sirolimus is related to the fact that it is a well-known agent that has been used for many years for other indications,^70–83^ (Supplementary Table S11). For instance, sirolimus or sirolimus-related compounds have been employed for the treatment of kidney,^70,71^ cardiac,^72^ and liver^73^ transplantation, systemic lupus erythematosus,^74^ autoimmune cytopenia,^75^ lymphangioleiomyomatosis,^76^ tuberous sclerosis complex,^77^ recurrent meningioma,^78^ pancreatic neuroendocrine tumors,^79^ advanced differentiated thyroid cancers,^80^ advanced breast cancer,^81^ β-cell lymphomas,^82^ and metastatic renal cell carcinoma.^83^

With respect to the clinical management of β-thalassemia patients, sirolimus was considered a repurposed drug when the first evidence of its ability to induce *in vitro* HbF production in ErPCs from β-thalassemia and SCD patients was reported and further validated.^28–31^

A second important conclusion of our trial is that sirolimus affects erythropoiesis and reduces the biochemical markers associated with ineffective erythropoiesis. For instance, ErPCs from sirolimus-treated patients exhibit a clear reduction in excess free α-globin chains. Targeting the network(s) regulating this parameter might be very important since in β-thalassemia, accumulated free α-globin forms intracellular precipitates that impair erythroid cell maturation and viability. Induction of autophagy is expected to help activate quality control systems that are able to mitigate β-thalassemia pathophysiology by degrading toxic free α-globin.^59,84,85^ The effects of autophagy-associated parameters will be the subject of a subsequent study.

In addition to reducing the accumulation of free α-globin, sirolimus treatment was able to modify other biochemical parameters associated with ineffective erythropoiesis, such as bilirubin, sTFR, and ferritin. All these parameters were higher in patients with β-thalassemia than in the unaffected population. We can speculate that the change in ferritin levels could be regarded as an indication of the modification of iron metabolism in its complex network with erythropoietic activity.^86,87^ The sirolimus-mediated reduction in these biochemical parameters might indicate an improvement in ineffective erythropoiesis. Moreover, these effects of sirolimus appeared late in treatment (starting from V8).

These data suggest that additional effects of sirolimus might occur *in vivo*, in addition to the stimulation of γ-globin gene expression and HbF production.

In terms of clinical data, transfusion burden was evaluated as an index of transfusion demand since it was adjusted for the pre-transfusion Hb concentration. No patient showed a major reduction in transfusion burden, but in the sirolimus-treated cohort, majority of the patients (five of the eight patients) displayed a significant reduction in the index of transfusion demand (*p* = 0.0056), in the first 6 months of therapy.

At this point, it is necessary to re-iterate that this pilot trial was designed to verify whether positive effects of sirolimus could be seen in β-thalassemia patients without altering their transfusion regimen or using low doses of sirolimus.

As far as the first point is concerned, it would be very interesting to test sirolimus on NTDT patients and on β-thalassemia patients who do not regularly receive blood transfusions. However, the data presented in this article should be considered together with the results obtained during the trial NCT04247750, which was focused on patients carrying the β^0^/β^0^ and β^+^/β^0^ thalassemia genotypes.

Second, we have to point out that the sirolimus content in the blood was variable and low. With regard to variability, the values observed cannot be considered unexpected since high variability has been found in clinical trials carried out for other diseases as well.^88–90^ The overall blood content was found to be lower than that expected from published data on other pathologies (Figure 3 and Table 4).^88–90^ For instance, in 90 patients with lymphangioleiomyomatosis, the average blood sirolimus level was 7.2 ± 2.6 ng/mL (range: 1.5–18.6 ng/mL) in the first year of sirolimus treatment under the average dose of 1.27 ± 0.47 mg/day (range: 0.5–2 mg/day).^89^ In our patients, the actual average dose was 1.2 mg/day at 3 months and 1.7 mg/day at 6 months. This sirolimus dosage was associated with a blood concentration of 2.2 ± 0.9 ng/mL after 3 months of treatment (range 1.1–4.5 ng/mL) and 3.0 ± 1.6 ng/mL after 6 months of treatment (range 1.0–4.6 ng/mL). When these data were compared with the HPLC and RT-qPCR data, it was concluded that these low levels of sirolimus were sufficient to cause changes in γ-globin gene expression.

Under these circumstances, the drug was well tolerated, without causing an alteration of the immunophenotype. Although no major side effects were observed, five patients exhibited stomatitis (for three of them, this effect was recurrent). Our study demonstrates that this was the only relevant side effect of the treatment, as reported in other cohorts of patients affected by other pathologies (tuberous sclerosis complex, lymphangioleiomyomatosis, organ transplantation, and cancer), who were treated with mTOR inhibitors.^65^ This issue should be considered in cases in which other sirolimus-based clinical interventions have been proposed. In this context, good oral hygiene should be maintained,^66^ and spicy, acidic, hot, or hard foods should be avoided. In addition, topical high-potency corticosteroids (e.g. clobetasol and dexamethasone), non-steroidal anti-inflammatory drugs (e.g. amlexanox paste), and anesthetics (e.g. viscous lidocaine) can be considered. The issues related to the possible side effects of mTOR inhibitors have been reviewed extensively elsewhere.^65–68^ Alternatively, future experimental trials should consider the use of other rapalogs.

This pilot study generated a small biobank of biological samples that could be useful for further studies and for the design of future clinical trials. For instance, the issue of sirolimus-activated autophagy might be of interest since this might explain the decrease in the excess of free α-globin chains in ErPCs from sirolimus-treated patients, as suggested by Lechauve *et al.*^59^ Moreover, a more complete study on the effects of sirolimus on the expression of all the genes belonging to the α-like and β-like globin gene cluster can be designed, including the analysis of the content of adult δ-globin mRNA and embryonic ζ- and ε-globin mRNAs. This could be of interest since we cannot exclude the effect of sirolimus on these genes. The impact of modulating the expression of embryonic globin genes is growingly being considered in the development of protocols for thalassemia treatment.^91,92^ These data will be considered together with the expression of known γ-globin gene repressors (e.g. BCL11A, MYB, LYAR, and ZBTB7A).^93–96^ Preliminary data on BCL11A and LYAR downregulation were obtained using ErPCs isolated from sirolimus-treated patients (unpublished data from Cristina Zuccato). In addition, we were able to study the effects of sirolimus on the biological activity of memory T cells (Figure 9) when stimulated by CEF peptides (mimicking sequences of the proteins of cytomegalovirus, Epstein–Barr virus, and Influenza A virus). This study might help to understand whether sirolimus could be effective in sustaining the long-term effects of vaccination, as recently reported^97,98^ (unpublished data from Matteo Zurlo). Finally, when the results of the THALA-RAP trial (NCT04247750, focusing on β^0^/β^0^ and β^0^/β^+^ thalassemia patients) are available, we might relate genotypes with the effects on HbF production and transfusion demand. This is not only limited to thalassemia mutations but also to HbF-related gene polymorphisms. In this context, it is intriguing to underline that patient n.22 carries an XmnI(−/+) polymorphism (associated with high HbF expression) and exhibited the best response in terms of reduction of transfusion demand. A good response to sirolimus was also demonstrated by the γ-globin mRNA increase in the blood and when ErPCs were analyzed (Figures 4 and 5).

In conclusion, despite the limited number of patients and the variability of response, the data indicate that low doses of sirolimus modify hematopoiesis and induce increased expression of γ-globin genes in a subset of β-thalassemia patients. Further clinical trials are warranted, and these could consider the possibility of testing the drug (a) in patients whose ErPCs do not respond to sirolimus and (b) in patients with less severe forms of the disease (for instance, those affected by NTDT). Future clinical trials should consider exploring the combination therapies and other end-points not considered in this trial. Furthermore, our study demonstrated that a 6-month-period is sufficient for detecting the sirolimus response.

## Supplemental Material

sj-pdf-1-tah-10.1177_20406207221100648 – Supplemental material for Expression of γ-globin genes in β-thalassemia patients treated with sirolimus: results from a pilot clinical trial (Sirthalaclin) Click here for additional data file. Supplemental material, sj-pdf-1-tah-10.1177_20406207221100648 for Expression of γ-globin genes in β-thalassemia patients treated with sirolimus: results from a pilot clinical trial (Sirthalaclin) by Cristina Zuccato, Lucia Carmela Cosenza, Matteo Zurlo, Jessica Gasparello, Chiara Papi, Elisabetta D’Aversa, Giulia Breveglieri, Ilaria Lampronti, Alessia Finotti, Monica Borgatti, Chiara Scapoli, Alice Stievano, Monica Fortini, Eric Ramazzotti, Nicola Marchetti, Marco Prosdocimi, Maria Rita Gamberini and Roberto Gambari in Therapeutic Advances in Hematology
