# Supplementary material for: Expression of γ-globin genes in β-thalassemia patients treated with sirolimus: results from a pilot clinical trial (Sirthalaclin)
Source: Ther Adv Hematol. 2022 Jun 21;13:20406207221100648. doi: 10.1177/20406207221100648 (PMC9218916; doi:10.1177/20406207221100648)
Supplement: sj-pdf-1-tah-10.1177_20406207221100648 – Supplemental material for Expression of γ-globin genes in β-thalassemia patients treated with sirolimus: results from a pilot clinical trial (Sirthalaclin) [file sj-pdf-1-tah-10.1177_20406207221100648.pdf]

Expression of γ-globin genes in β-thalassemia patients treated with sirolimus: results from a pilot clinical trial (Sirthalaclin)

Cristina Zuccato[1,*], Lucia Carmela Cosenza[1,*], Matteo Zurlo[1,*], Jessica Gasparello[1], Chiara Papi[1], Elisabetta D'Aversa[1], Giulia Breveglieri[1], Ilaria Lampronti[1,2], Alessia Finotti[1,2], Monica Borgatti[1,2], Chiara Scapoli[3], Alice Stievano[5], Monica Fortini[4], Eric Ramazzotti[5], Nicola Marchetti[6], Marco Prosdocimi[7], Maria Rita Gamberini[4] and Roberto Gambari[1,2]

[1]Dipartimento di Scienze della Vita e Biotecnologie, Sezione di Biochimica e Biologia Molecolare, Università degli Studi di Ferrara, Ferrara, Italy;
[2]Thal-LAB, Laboratorio di Ricerca Elio Zago sulla Terapia Farmacologica e Farmacogenomica della Talassemia, Università degli Studi di Ferrara, Ferrara, Italy;
[3]Dipartimento di Scienze della Vita e Biotecnologie, Sezione di Biologia ed Evoluzione, Università degli Studi di Ferrara, Ferrara, Italy;
[4]Unità Operativa Interdipartimentale di Day Hospital della Talassemia e delle Emoglobinopatie, Arcispedale S. Anna di Ferrara, Ferrara, Italy;
[5]Laboratorio Unico Metropolitano, Ospedale Maggiore, Azienda USL di Bologna, Bologna, Italy;
[6]Dipartimento di Scienze Chimiche, Farmaceutiche e Agrarie, Università degli Studi di Ferrara, Ferrara, Italy;
[7]Rare Partners S.r.L. Impresa Sociale, Milano, Italy;
[8]Center "Chiara Gemmo and Elio Zago" for the Research on Thalassemia.

Corresponding authors:

Roberto Gambari, Dipartimento di Scienze della Vita e Biotecnologie, Sezione di Biochimica e Biologia Molecolare, Università degli Studi di Ferrara, Ferrara, Italy
gam@unife.it

Maria Rita Gamberini, Unità Operativa Interdipartimentale di Day Hospital della Talassemia e delle Emoglobinopatie, Arcispedale S. Anna di Ferrara, Ferrara, Italy
m.gamberini@ospfe.it

(*) Co-first authors

SUPPLEMENTARY MATERIAL

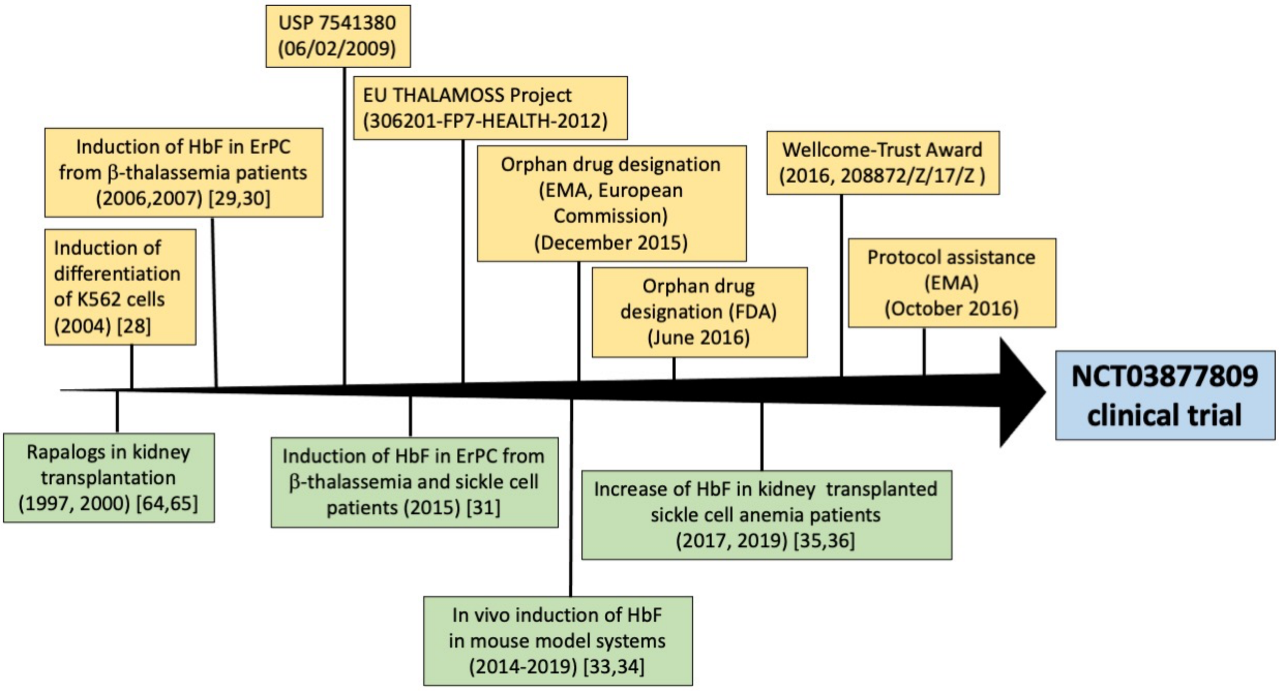

Figure S1. Summary of the laboratory research achievements and technological transfer activity leading to the design of the NCT03877809 trial. Yellow-boxed are activities of our group; green-boxed are key studies from the literature on sirolimus and other rapalogs.

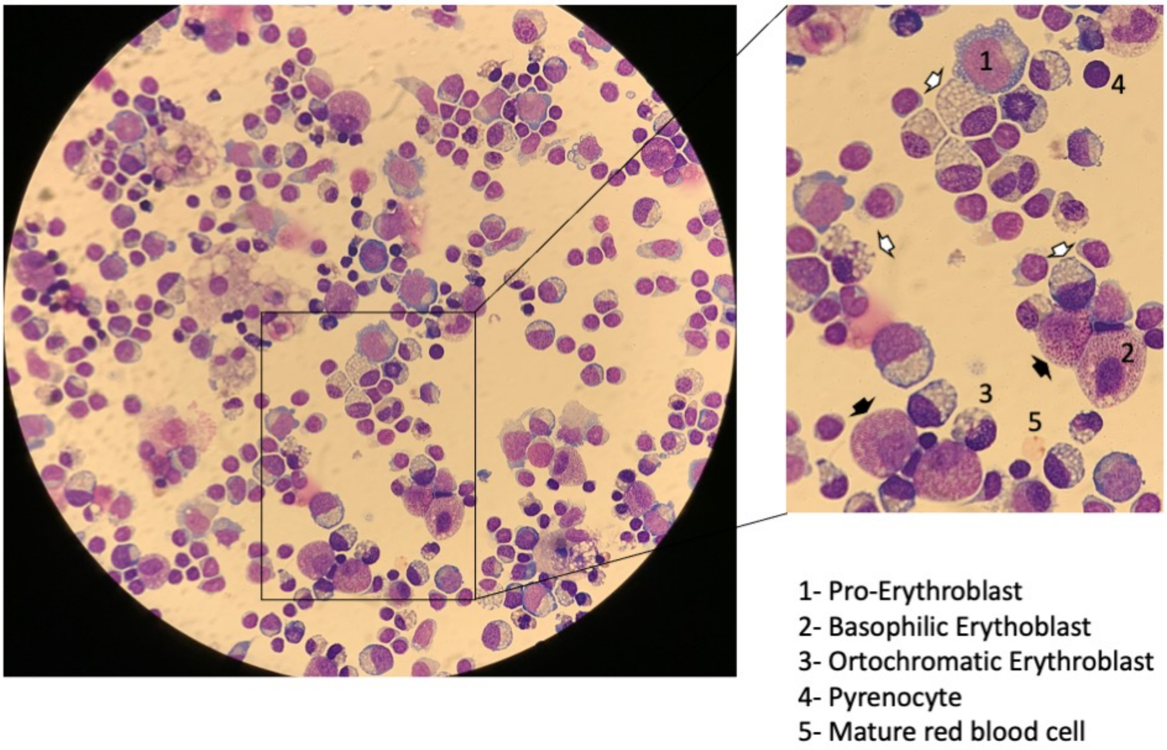

Figure S2. Representative cytospin image from cultured ErPCs from a β-Thalassemia patient. May-Grunwald Giemsa staining was performed. In this picture, almost all the stages of erythroid differentiation are clearly visible, ranging from the pro-erythroblast to the mature red blood cells and pyrenocytes. Some common alterations of β-Thalassemia are evident like high cellular vacuolization (white arrows) and basophilic stippling (black arrows).

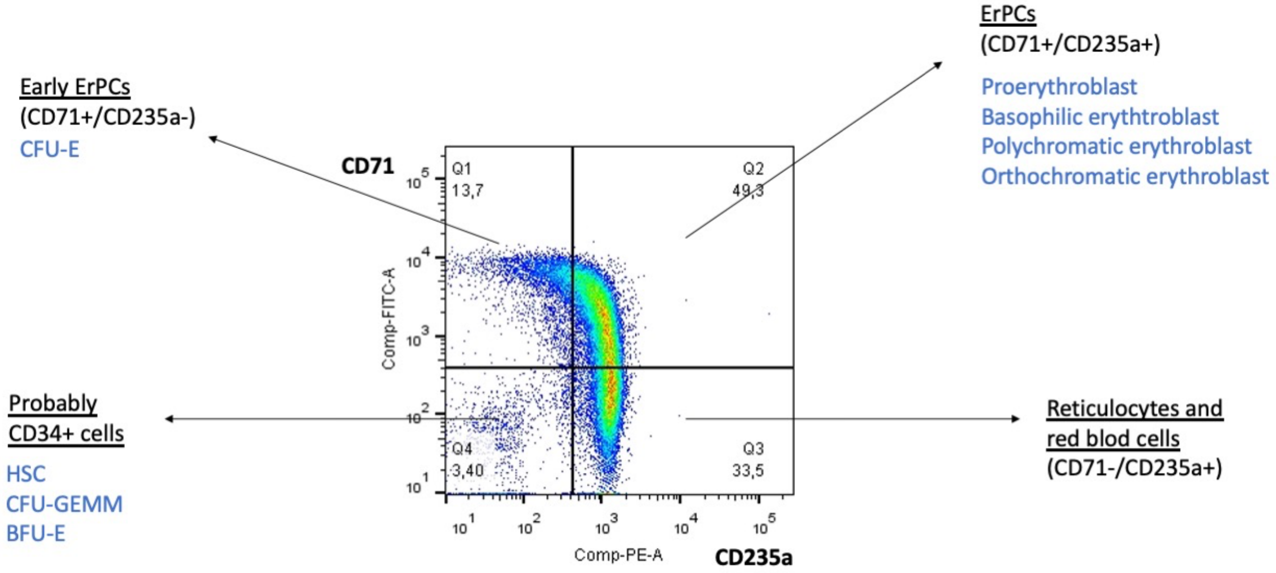

Figure S3. Representative bidimensional dot plot obtained from cultured ErPCs following two weeks of erythropoietin stimulation. Q1 (upper left quadrant): cells positive to CD71 marker only (early erythroid precursors). Q2 (upper right quadrant): more mature ErPCs, ranging from the pro-erythroblast to orthochromatic erythroblast (in all these stages of differentiation the cells displays both erythropoiesis marker CD71 and CD235a). Q3 (lower right quadrant): reticulocytes and mature red blood cells (positive only to CD235a). Q4 (lower left quadrant): small portion of cells negative to both markers (probably CD34^{+} cells that have not yet begun to differentiate).

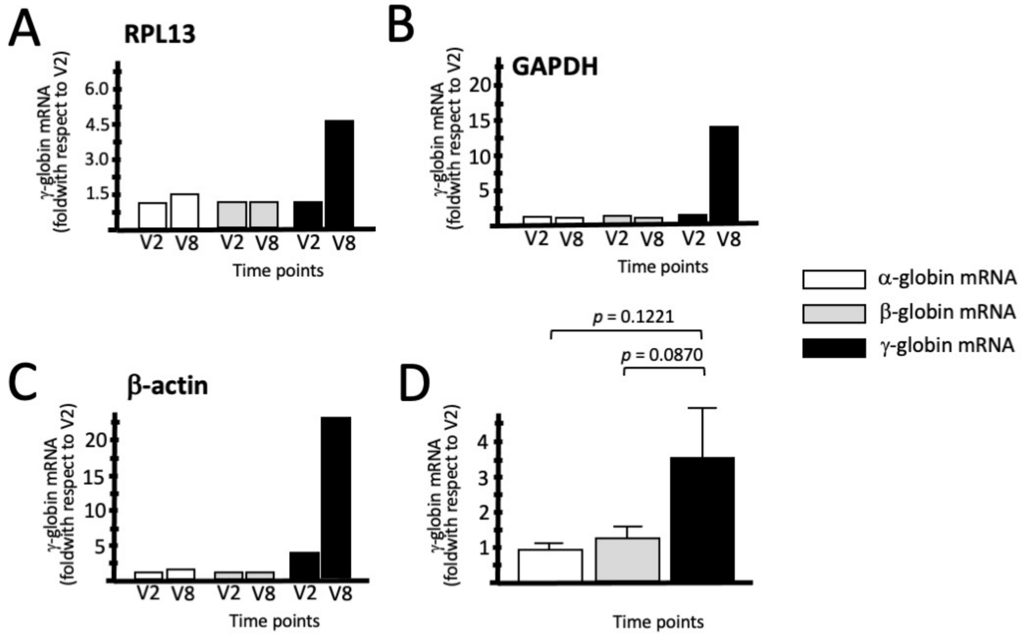

Figure S4. Content of α-globin, β-globin and γ-globin mRNAs in the blood of patients treated with sirolimus. A-C. Representative data obtained with the blood samples of patient n.11 using RPL13A (A), GAPDH (B) and β-actin (C) control sequences, as indicated. α-globin mRNA: white bars; β-globin: gray bars; γ-globin mRNAs: black bars. D. Content of α-globin, β-globin and γ-globin mRNAs in the blood of patients treated with sirolimus. The data represent the fold values with respect to V2.

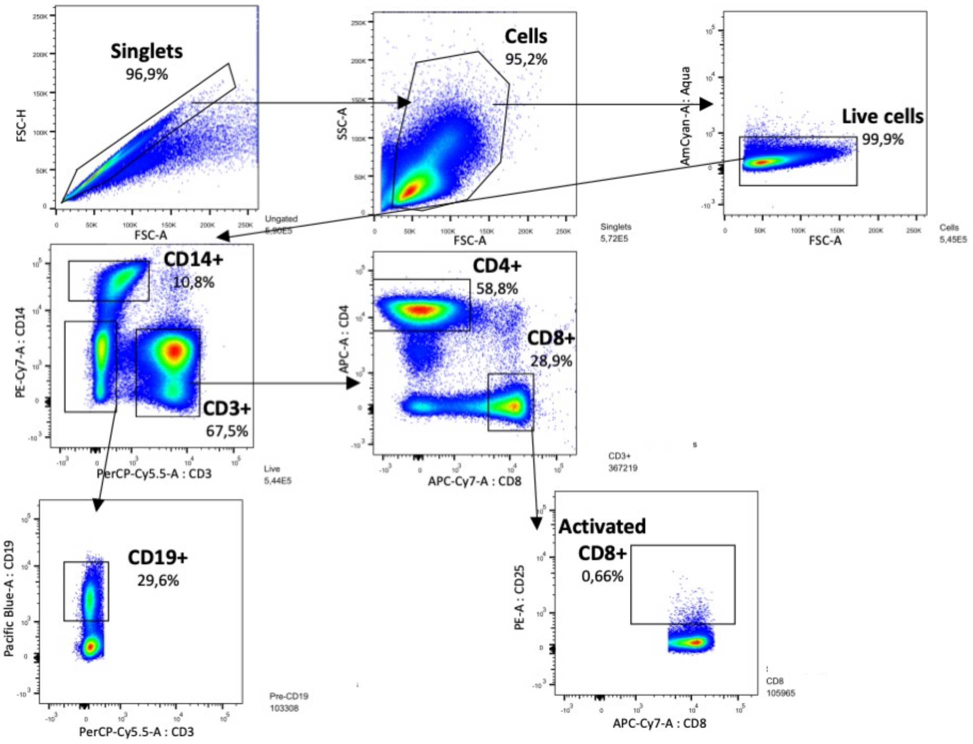

Figure S5A. Immunophenotype: V2. The adopted gating strategy allows to discriminate single cells (FSC-H vs FSC-A) from clumped cells, within these we are further able to distinguish single cells from debris (SSC-A vs FSC-A). A tracker was also used to exclude dead cells (Aqua Dead Cell Stain kit) in order to have the maximum precision in analyzing only live cells after thawing. With this panel of antibodies, we were able to subdivide PBMCs population into monocytes ($CD14^{+}$), B cells ($CD14^{-}/CD3^{-}/CD19^{+}$), T cells ($CD14^{-}/CD3^{+}$), $CD4^{+}$ T cells ($CD14^{-}/CD3^{+}/CD4^{+}$), $CD8^{+}$ T cells ($CD14^{-}/CD3^{+}/CD8^{+}$) and $CD8^{+}$ Activated T cells ($CD14^{-}/CD3^{+}/CD8^{+}/CD25^{+}$). Representative FACS data performed using samples from patient n.18 (V2).

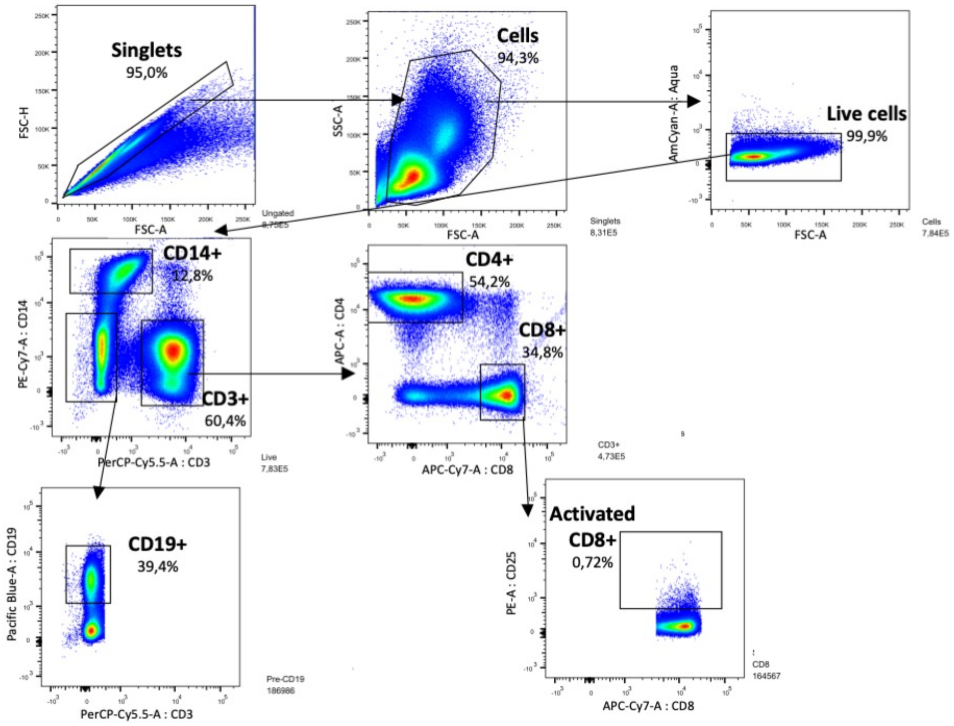

Figure S5B. Immunophenotype: V6. For technical detail, see the legend to Figure 5A. Representative FACS data performed using samples from patient n.18 (V6, after three months therapy with sirolimus).

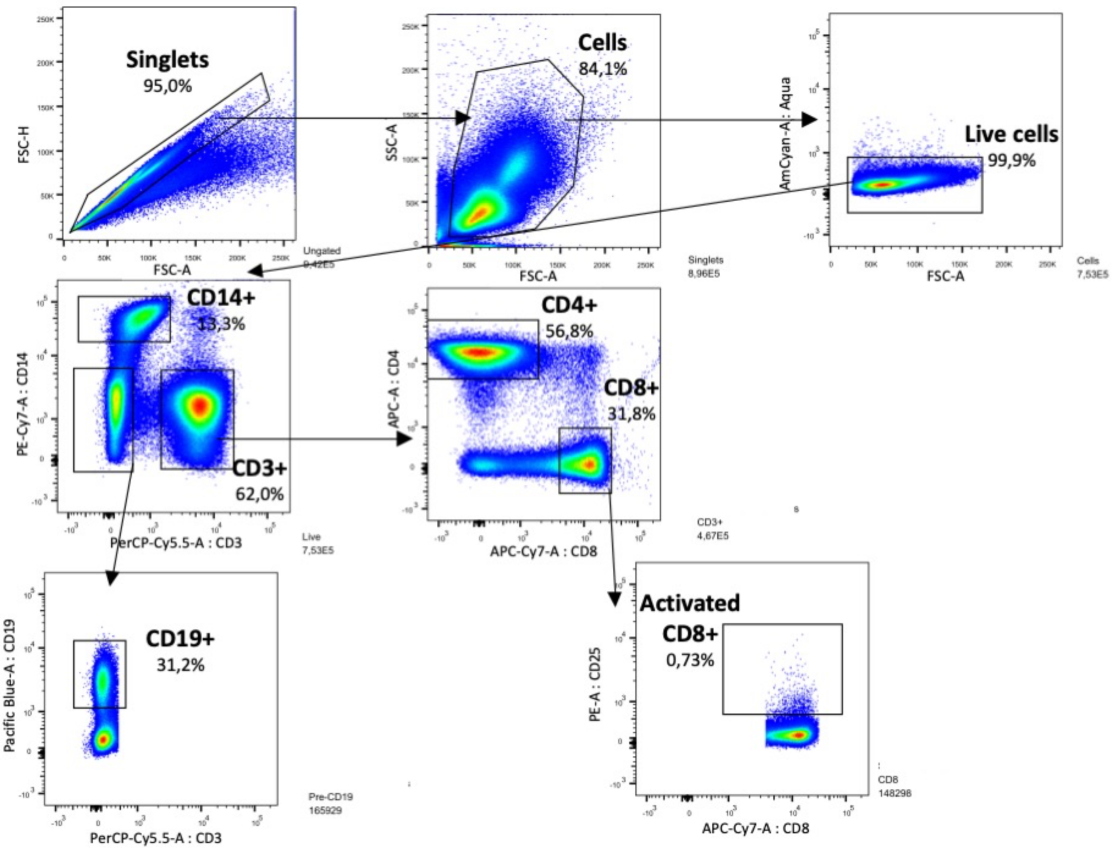

Figure S5C. Immunophenotype: V8. For technical detail, see the legend to Figure 5A. Representative FACS data performed using samples from patient n.18 (V8, after six months therapy with sirolimus).

**TABLE S1. Inclusion criteria**	
**Age**	Patients with age over 18 years
**Genotype**	Patients with confirmed genotypes of homozygous/compound heterozygous β-thalassaemia major (β+/β+, β+/β0 thalassemia genotype)
**Clinical parameters**	Patients with documented diagnosis of transfusion dependence requiring more than 8 transfusions during the preceding 12 months
	Patients on regular transfusion since at least 6 years
	Patients with splenectomy performed at least 60 days before selection or spleen dimensions < 20 cm in the longitudinal diameter as detected by abdominal echography
**Management**	A medically reliable method of contraception for the entire study duration must be counseled for female participants potential childbearing
**Ethical/Regulatory issues**	Patients willing to follow all the study requirements and perform all the study visits and to cooperate with the investigator
	Patient able to understand the informed consent and to sign it before any study procedure

**TABLE S2. Exclusion criteria**	
**Analytical clinical parameters**	White blood cell count <3000 cells/μl and/or Granulocytes <1500/μl
	Platelet count <150.000/uL and >1 x10^6 /μl
	Dyslipidemia (total cholesterol > 240 mg/dl; triglycerides > 200 mg/dl)
	Significant proteinuria (>1g/24 hrs)
	Increased levels of transaminases (more than 3 times the upper limit of normal)
	Coexisting viral infections (positivity for human immunodeficiency virus (HIV) antibody; active hepatitis B (HBV) or hepatitis C (HCV) as demonstrated by the presence of hepatitis B surface antigen (HBsAg) and a positive HCV-RNA test, HBcAb and HBV-DNA positivity)
**Management/Therapy**	Patients treated with hydroxyurea at selection visit or in the last 6 months
	Ongoing treatment with drugs possibly affecting sirolimus actions, including treatment with Macrolidic antibiotics (chlarithromycin)
	Cytotoxic agents, systemic corticosteroids, immunosuppressants or anticoagulant therapy such as warfarin or heparin within 28 days before inclusion (prophylactic aspirin up to 100 mg / day is allowed)
	Iron chelation therapy: Deferiprone is not accepted as a chelation therapy drug, while Desferioxamine and Deferasirox are allowed
	Subject with any significant medical condition and/or laboratory abnormality considered by the investigator as not adequately controlled at the time of selection
	Treatment with live vaccines within 90 days preceding the selection
**Previous clinical issues**	History of severe allergic or anaphylactic reactions or hypersensitivity to excipients in the experimental drug
	Cardiovascular complications (heart failure as classified by the New York Heart Association (NYHA) classification 3 or higher; uncontrolled hypertension defined as systolic blood pressure (BP) ≥ 140 mm Hg or diastolic BP ≥ 90 mm Hg; significant arrhythmia requiring treatment; QTc> 450 msec on selection ECG; ejection fraction <50% by echocardiogram, MUGA or cardiac magnetic resonance; myocardial infarction within 6 months prior of selection
	Major surgery (including splenectomy) within 60 days before selection (patients must have fully recovered from any previous surgery)
	Subject with history or current malignancies (solid tumors and hematological malignancies) or presence of masses/tumor detected by ultrasound at selection
**Pregnancy related issues**	Pregnant or lactating women; patients who are expecting to get pregnant during the next 12 months

TABLE S3. CONSORT 2010 checklist of information included			
**Section/Topic**	**Item No**	**Checklist item**	**Reported on page No**
**Title and abstract**			
	1a	Identification as a pilot or feasibility randomised trial in the title	Page 1 (*)
	1b	Structured summary of pilot trial design, methods, results, and conclusions (for specific guidance see CONSORT abstract extension for pilot trials)	Page 2
**Introduction**			
Background and objectives	2a	Scientific background and explanation of rationale for future definitive trial	Pages 4-6
	2b	Specific objectives or research questions for pilot trial	Pages 5 and 6
**Methods**			
Trial design	3a	Description of pilot trial design (such as parallel, factorial)	Page 7
	3b	Important changes to methods after pilot trial commencement (such as eligibility criteria), with reasons	N.A.
Participants	4a	Eligibility criteria for participants	Supplementary materials, page 4
	4b	Settings and locations where the data were collected	Page 7
	4c	How participants were identified and consented	Pages 7 and 15
Interventions	5	The interventions for each group with sufficient details to allow replication, including how and when they were actually administered	Pages 7 and 8
Outcomes	6a	Completely defined prespecified assessments or measurements to address each pilot trial objective specified in 2b, including how and when they were assessed	Pages 17-25
	6b	Any changes to pilot trial assessments or measurements after the pilot trial commenced, with reasons	N.A.
	6c	If applicable, prespecified criteria used to judge whether, or how, to proceed with future definitive trial	Page 26
Sample size	7a	Rationale for numbers in the pilot trial	Pages 15,16 and 26

	7b	When applicable, explanation of any interim analyses	Page 7
Randomisation:			
Sequence generation	8a	Method used to generate the random allocation sequence	N.A.
	8b	Type of randomisation(s); details of any restriction (such as blocking and block size)	N.A:
Allocation concealment mechanism	9	Mechanism used to implement the random allocation sequence (such as sequentially numbered containers), describing any steps taken to conceal the sequence until interventions were assigned	N.A.
Implementation	10	Who generated the random allocation sequence, who enrolled participants, and who assigned participants to interventions	N.A.
Blinding	11a	If done, who was blinded after assignment to interventions (for example, participants, care providers, those assessing outcomes) and how	N.A.
	11b	If relevant, description of the similarity of interventions	N.A.
Statistical methods	12	Methods used to address each pilot trial objective whether qualitative or quantitative	Pages 7-14
**Results**			
Participant flow (a diagram is strongly recommended)	13a	For each group, the numbers of participants who were approached and/or assessed for eligibility, received intended treatment, and were assessed for each objective	Page 15
	13b	For each group, losses and exclusions, together with reasons	N.A. (**)
Recruitment	14a	Dates defining the periods of recruitment and follow-up	Page 7
	14b	Why the pilot trial ended or was stopped	N.A.
Baseline data	15	A table showing baseline demographic and clinical characteristics for each group	Supplementary materials, page 10
Numbers analysed	16	For each objective, number of participants (denominator) included in each analysis.	Pages 16 and 17
Outcomes and estimation	17	For each objective, results including expressions of uncertainty (such as 95% confidence interval) for any estimates.	Pages 15-25
Ancillary analyses	18	Results of any other analyses performed that could be used to inform the future definitive trial	Pages 31 and 32

Harms	19	All important harms or unintended effects in each group	Page 30
	19a	If relevant, other important unintended consequences	Page 30
**Discussion**			
Limitations	20	Pilot trial limitations, addressing sources of potential bias and remaining uncertainty about feasibility	Page 32
Generalisability	21	Generalisability (applicability) of pilot trial methods and findings to future definitive trial and other studies	Page 31 and 32
Interpretation	22	Interpretation consistent with pilot trial objectives and findings, balancing potential benefits and harms, and considering other relevant evidence	Pages 26-29
	22a	Implications for progression from pilot to future definitive trial, including any proposed amendments	Pages 31 and 32
**Other information**			
Registration	23	Registration number for pilot trial and name of trial registry	Pages 5 and 7
Protocol	24	Where the pilot trial protocol can be accessed, if available	Page 5
Funding	25	Sources of funding and other support (such as supply of drugs), role of funders	Page 33
	26	Ethical approval or approval by research review committee, confirmed with reference number	Page 7

(*) = unless otherwise stated, the number of pages refers to the main text.
(**) = none of the patients had to stop sirolimus for safety reason.
N.A. = not applicable

TABLE S4. Clinical data of recruited β-thalassemia patients at time of inclusion (V2)								
	**Patient n.1**	**Patient n.2**	**Patient n.10**	**Patient n.11**	**Patient n.18**	**Patient n.21**	**Patient n.22**	**Patient n.24**
Average pre-transfusion Hb (g/dl)	9.50	10.40	10.40	9.90	9.80	10.01	9.12	9.10
Red cell consumptions (ml/kg/year)	126.44	111.91	100.51	99.19	104.62	94.35	131.49	114.58
White Bood Cells (10^3/µl) **(<3000 cells/µl)**	12.50	6.71	17.91	13.94	12.54	8.17	8.98	5.80
Neutrophil Granulocytes x 10^3/µl (**<1500/ µl**)	6.58	3.49	6.41	7.92	4.57	4.02	4.91	3.44
Platelets x 10^3/µl (**<150.000/ul or >1 x10^6/ul**)	473	315	443	570	355	803	396	259
Cholesterol (mg/dl) (**> 240 mg/dl**)	189	147	139	164	125	168	121	94
Triglycerides (mg/dl) (**> 200 mg/dl**)	102	105	76	195	52	139	53	146
Proteinuria (mg/ 24hrs) (**> 1g/24 hrs**)	307	74	113	156	104	313	136	185
Serum creatinine (mg/dl)	0.54	0.79	0.69	0.61	0.80	0.75	0.57	1.02
Glycemia (mg/dl)	77	113	103	86	93	89	83	99
Serum albumin (g/dl)	4.34	4.47	4.35	4.41	4.19	4.46	4.18	4.65
Transaminases (ALT-AST; UI/L)° (**< 3 times the upper limit of normal**)	36-26	17-21	20-21	12-17	27-23	19-20	14-18	28-24
Endocrine complications: ^								
Hypogonadism	yes	yes	no	yes	yes	yes	no	no
Hypothyroidism	no	no	no	no	no	yes	no	no
Hypoparathyroidism	no	no	yes	no	no	no	no	no
Diabetes mellitus	no	no	no	no	no	no	no	no
Osteoporosis	no	yes	no	yes	yes	no	yes	no
Iron chelation therapy: ^^	DFX	DFX	DFO/DFX**	DFX	DFO/DFX**	DFX	DFX	DFX
Iron accumulation in liver and cardiac tissues (MRI-T2*)								
Global cardiac T2* (ms)+	37	37	42	41	36	35	34	37
Liver iron concentration (LIC)++ (mg(Fe)/g dry liver tissue)	4.06	8.14	1.79	4.13	1.16	4.80	6.15	4.21

(*) Inclusion/Exclusion criteria are bolded in parenthesis
(°) ALT and AST reference range: females < 35 UI/l; males < 50 UI/L
(^) Diagnosis performed according to TIF guidelines (44)
(^^) Desferioxamine= DFO; Deferasirox = DFX; (**) sequential regimen
(+) reference range: ≥20 ms normal vaue (no significant iron load)
(++) reference range: LIC <3mg/g: no significant iron load; LIC 3-7 mg/g: mild iron load; LIC≥7 and <15 mg/g: moderate iron load; LIC ≥15 mg/g: severe iron load

All patients were negative for:

- active infection with immunodeficiency virus (HIV), hepatitis B, hepatitis C;
- treatment with hydroxyurea at selection visit or in the last 6 months;
- treatment with macrolide antibiotics (chlarithromycin), cytotoxic agents, systemic corticosteroids, immunosuppressants or anticoagulants;
- injection of live vaccines within 90 days preceding the selection;
- severe allergic or anaphylactic reactions or hypersensitivity to excipients in the experimental drug;
- cardiovascular complications, uncontrolled hypertension, significant arrhythmia requiring treatment; myocardial infarction within 6 months prior of selection;
- major surgery within 60 days before selection;
- history or current malignancies or presence of masses/tumor detected by ultrasound at selection.

TABLE S5. Variation of blood γ-globin mRNA: relative γ-globin mRNA value (*)				
**Patient number**	**V2**	**V6**	**V8**	**V11**
1	12.01	21.02	18.02	(**)
2	7.62	4.36	15.47	8.61
10	17.71	38.78	50.83	48.80
11	1.00	14.05	13.85	(**)
18	3.26	6.72	12.26	(**)
21	1.49	1.94	0.60	(**)
22	7.04	9.15	30.27	(**)
24	16.31	17.94	13.66	48.59

(*) Values relative to patient n.11
(**) Patients 1, 11, 18, 21 and 22 concluded the trial at V8

TABLE S6. Variation of ErPC γ-globin mRNA: relative γ-globin mRNA value (*)				
**Patient number**	**V2**	**V6**	**V8**	**V11**
1	4.05	4.86	3.64	(**)
2	1.5	1.05	5.25	2.25
10	5.5	6.05	8.25	15.95
11	1.80	2.70	10.26	(**)
18	1.85	4.44	3.33	(**)
21	4.01	6.40	2.80	(**)
22	2.9	10.73	4.06	(**)
24	1	7.10	6.50	1.51

(*) Values relative to patient n.24
(**) Patients 1, 11, 18, 21 and 22 concluded the trial at V8

TABLE S7. Variation of total bilirubin levels (mg/dl) (**)				
**Patient number**	**V2**	**V6**	**V8**	**V11**
1	2.89	3.63	2.71	(*)
2	1.81	1.23	1.27	1.27
10	2.43	1.66	1.70	1.53
11	2.61	2.25	2.30	(*)
18	1.17	1.37	0.90	(*)
21	1.88	1.55	1.59	(*)
22	1.12	0.98	1.04	(*)
24	2.68	1.92	1.62	1.86

(*) Patients 1, 11, 18, 21 and 22 concluded the trial at V8
(**) Reference range: <1.2mg/dl

TABLE S8. Variation of soluble transferrin receptor levels (mg/l) (**)				
**Patient number**	**V2**	**V6**	**V8**	**V11**
1	2.51	2.71	2.31	(*)
2	2.52	2.12	1.94	1.75
10	2.33	3.15	1.95	1.82
11	2.85	3.47	3.05	(*)
18	2.48	2.37	2.08	(*)
21	3.58	2.87	2.95	(*)
22	1.79	1.84	1.86	(*)
24	3.94	2.71	2.60	3.48

(*) Patients 1, 11, 18, 21 and 22 concluded the trial at V8
(**) Reference range: 0.9-2.01 mg/l

TABLE S9. Variation of ferritin levels (ng/ml) (**)				
**Patient number**	**V2**	**V6**	**V8**	**V11**
1	826	908	631	(*)
2	1110	1191	700	779
10	842	546	499	488
11	608	357	365	(*)
18	552	545	414	(*)
21	767	598	552	(*)
22	542	504	477	(*)
24	1221	1213	1191	1146

(*) Patients 1, 11, 18, 21 and 22 concluded the trial at V8.
(**) Reference range: 11-306 ng/ml in females; 24-336 ng/ml in males

**TABLE S10. Characteristics of stomatitis occurring during the trial** (*)	
Number of patients (%)	5/8 (62.5%)
Number of episodes	11
Patients with recurrent episodes (≥2)	3
Episode duration in days, median (range)	7.5 (1-22)
Time to the first stomatitis events in days, median (range)	96 (31-190)
Severity (grade) (**)	Mild: 5<br>Moderate: 6

(*) Stomatitis or oral mucositis was defined as a disorder characterized by ulceration or inflammation of the oral mucosa. Symptoms and clinical examination: pain, superficial location, single or multiple ulcers, location in the inner lip, tongue and soft palate (dimension generally <1 cm in diameter, grayish-white colored and surrounded by an erythematous margin).
(**) From Common Terminology Criteria for Adverse Events (CTCAE) -Version 5.0 (November 27, 2017). Grade 1: asymptomatic or mild symptoms; intervention not indicated; Grade 2: moderate pain or ulcer that does not interfere with oral intake; modified diet indicated; Grade 3: severe pain; interfering with oral intake; Grade 4: life-threatening consequences; urgent intervention indicated.

TABLE S11. Examples of clinical studies using mTOR inhibitors		
**Clinical application**	**Drug**	**Major conclusions**
Organ transplantation: Kidney	Sirolimus; everolimus	Issues covered: (a) prevention of immune dysfunction and renal function preservation in de novo renal transplantation; (b) chronic dysfunction of the renal graft; (c) cardiovascular effects; (d) de novo post-transplant diabetes, and (e) de novo tumor pathology (Kahan, 1997 [64]; Vasquez, 2000 [65]).
Organ transplantation: Cardiac interventions	Everolimus	Everolimus may reduce late term complications, including coronary allograft vasculopathy (CAV), while maintaining the low cellular rejection rates seen with standard therapy (Schaffer and Ross, 2010 [66]).
Organ transplantation: Liver	Everolimus	Everolimus combined with low-dose calcineurin inhibitors (CNIs) decreases the risk acute rejection (Tang et al., 2015 [67]).
Lupus erythematosus (SLE)	Sirolimus	Sirolimus was found promising and well-tolerated (Ji et al., 2020 [68]).
Autoimmune cytopenias	Sirolimus	Sirolimus was effective for patients with primary relapsed/refractory autoimmune cytopenia with a low relapse rate and good tolerance (Li et al., 2020 [69]).
Lymphangioleiomyomatosis (LAM)	Sirolimus; everolimus	Sirolimus and everolimus were recommended for the treatment of LAM because they could stabilize lung function and alleviate renal AML (Wang et al., 2020 [70]).
Tuberous sclerosis complex	Sirolimus; Everolimus	Orally administered everolimus significantly reduced the size of sub-ependymal giant cell astrocytoma and renal angiomyolipoma (Sasongko et al., 2016 [71]).
Cancer: Recurrent meningioma	Everolimus	The combination of everolimus and octreotide was associated with clinical and radiological activity in aggressive meningiomas and warrants further studies (Graillon et al., 2020 [72]).
Cancer: Pancreatic neuroendocrine tumours (NET)	Everolimus	Everolimus should be recommended as the first line therapy for patients with symptomatic malignant unresectable insulin-secreting pNETs, to control the endocrine syndrome regardless of tumor growth (Gallo et al., 2017 [73]).
Cancer: Advanced Differentiated Thyroid Cancers	Sirolimus	The combination of sirolimus, with a well-known cytotoxic agent, cyclophosphamide, provides a well-tolerated and promising alternative treatment for advanced, differentiated thyroid cancers (Manohar et al., 2015 [74]).
Cancer: Advanced breast cancer	Everolimus	Everolimus, in combination with exemestane, is proposed for patients with advanced hormone receptor-positive/HER2-negative breast cancer (Hortobagyi, 2015 [75]).
Cancer: B-cell lymphomas	Everolimus	A large study on relapsed/refractory diffuse large B-cell lymphoma (DLBCL) confirmed the substantial activity and good tolerability of everolimus, with thrombocytopenia being the main toxicity. The combination of everolimus and rituximab showed encouraging results, without increasing toxicity (Merli et al., 2015 [76]).
Cancer: Metastatic renal cell carcinoma	Everolimus	A Phase III trial demonstrated superiority at interim analysis for everolimus over placebo in patients with metastatic renal cell carcinoma (mRCC) (Motzer et al., 2010 [77]).

Supplementary Methods

Cytospin and morphological analysis of ErPCs.

Briefly, $1x10^5$ cells were concentrated in 100 ul of PBS and subsequently loaded through a special reservoir on a microscope slide by cytocentrifuge (5 min at 550 rpm), prepared slides are dried overnight under a chemical hood. The next day the glasses are covered with undiluted May Grunwald dye for 4 minutes (ethanol contained in this solution is sufficient to fix cells on the slide), then an equal volume of double distilled water is added on each glass and incubated another 4 minutes. At the end of the incubation, the glasses are drained and then covered with GIEMSA dye diluted 1:10 and incubated for other 12 minutes. Finally, the glasses are washed with abundant double-distilled water and dried overnight under a chemical hood. The prepared slides were then analyzed with a Nikon eclipse 80i optical microscope using a 40x and 60x oil immersion objective and captured with Nikon NIS Element imaging software.

Flow Cytometry based assessment of erythroid differentiation of in vitro cultured ErPCs.

In order to verify the expression of typical markers of erythropoiesis (CD71/d235a) by flow cytometry, 1 million cells in culture with erythropoietin for two weeks were collected. The cells were washed in PBS and subsequently labeled with 10 ul of antibody against CD71 (Miltenyi Biotec CD71-FITC cat.n.130-098-779) and 10 ul of antibody against CD235a (Miltenyi Biotec Glycophorin A-PE cat.n.130-100-259). After 15 min of incubation in the dark, cells are washed twice in PBS and resuspended in 200 µl of PBS prior to acquisition. Samples were acquired using a BD FACSCanto™ II Flow Cytometry system and obtained data analyzed using FlowJo v.10 software.

RT-qPCR analysis of content of α-globin, β-globin and γ-globin mRNAs

For gene expression analysis 500 ng of total RNA was reverse transcribed by using the TaqMan® Reverse Transcription Reagents and random hexamers (Applied Biosystems, Life Technologies, Carlsbad, CA, USA). Quantitative real time PCR assay, to quantify the expression of the globin genes, was carried out using two different reaction mixtures, the first one containing α, β, and γ-globin probe and primers, the second one containing GAPDH, RPL13A, β-actin probes and primers. The primers and probes used are listed in **Table I**.

TABLE I. Sequences of the primers and probes employed	
**Primer/probes**	**Sequence**
α-globin forward (primer)	5'-CGACAAGACCAACGTCAAGG-3'
α-globin reverse (primer)	5'-GGTCTTGGTGGTGGGGAAG-3'
α-globin probe	5'-HEX-ACATCCTCTCCAGGGCCTCCG-BFQ-3'
β-globin forward (primer)	5'-GGGCACCTTTGCCACAC-3'
β-globin reverse (primer)	5'-GGTGAATTCTTTGCCAAAGTGAT-3'
β-globin probe	5'-Cy5-ACGTTGCCCAGGAGCCTGAAG-BRQ-3'
γ-globin forward (primer)	5'-TGACAAGCTGCATGTGGATC-3'
γ-globin reverse (primer)	5'-TTCTTTGCCGAAATGGATTGC-3'
γ-globin probe	5'-FAM-TCACCAGCACATTTCCCAGGAGC-BFQ-3'
RPL13A forward (primer)	5'-GGCAATTTCTACAGAAACAAGTTG-3'
RPL13A reverse (primer)	5'-GTTTTGTGGGGCAGCATCC-3'
RPL13A probe	5'-HEX-CGCACGGTCCGCCAGAAGAT-BFQ-3'
β-actin forward (primer)	5'-ACAGAGCCTCGCCTTTG-3'
β-actin reverse (primer)	5'-ACGATGGAGGGGAAGACG-3'
β-actin probe	5'-Cy5-CCTTGCACATGCCGGAGCC-BRQ-3'
GAPDH forward (primer)	5'-ACATCGCTCAGACACCATG-3'
GAPDH reverse (primer)	5'-TGTAGTTGAGGTCAATGAAGGG-3'
GAPDH probe	5'-FAM-AAGGTCGGAGTCAACGGATTTGGTC-BFQ-3'

Each reaction mixture contained 1x TaKaRa Ex Taq® DNA Polymerase (Takara Bio Inc., Shiga, Japan), 500 nM forward and reverse primers and the 250 nM probes (Integrated DNA Technologies, Castenaso, Italy). The assays were carried out using CFX96 Touch Real-Time PCR System (Bio-Rad, Hercules, California, USA). After an initial denaturation at 95°C for 1 min, the reactions were performed for 50 cycles (95°C for 15 sec, 60°C for 60 sec). Data were analyzed by employing the CFX manager software (Bio-Rad, Hercules, California, USA). To compare gene expression of each template amplified, the ΔΔCt method was used [29,30].

Calculation methods for the transfusion indexes

The period of treatment from V2 to V8 lasts approximately 180 days. However, transfusion periods only approximately correspond to the scheduled visits because transfusions were not always performed in the same day of the visit. The first transfusion episode considered as performed under therapy began in the day of V2 or in the following days. A transfusion episode includes the day of transfusion and the interval up to the following transfusion. The last transfusion episode was completed within V8 or after no more than 3 days. The

transfusions performed in a period of about 180 days before V2 were considered for the estimate of the baseline blood consumption.

As an example:

Patient n.11	Duration (days)
**Treatment with sirolimus**	175
**Period of transfusion under sirolimus**	177
**Baseline period of transfusion**	167

At each transfusion, the pre-transfusion hemoglobin concentration was measured; the number of red cell concentrates transfused was recorded; the volume of pure red cells transfused was estimated from the volume and the hematocrit of the blood unit. The patients' body weight was measured regularly. Therefore, for each period of transfusion, the following parameters were calculated: (a) average pre-transfusion hemoglobin concentration (g/dL); (b) total number of red cell concentrates transfused in the period; (c) total volume of pure red cells transfused (mL); (d) average body weight (kg); (e) red cell consumption, in mL of pure red cells per kg body weight per year (this adjustment is necessary because the body weight may change from the baseline and the transfusion periods have slightly different durations); (f) index of transfusion demand is calculated dividing the red cell consumption by the average pre-transfusion hemoglobin concentration (this further adjustment is advisable because the average pre-transfusion hemoglobin concentration, too, may change from the baseline). If the endogenous production of hemoglobin increases during the treatment with sirolimus, then this parameter should decrease proportionally.
